# Neural networks with optimized single-neuron adaptation uncover biologically plausible regularization

**DOI:** 10.1371/journal.pcbi.1012567

**Published:** 2024-12-13

**Authors:** Victor Geadah, Stefan Horoi, Giancarlo Kerg, Guy Wolf, Guillaume Lajoie

**Affiliations:** 1 Program in Applied and Computational Mathematics, Princeton University, Princeton, New Jersey, United States of America; 2 Mila - Quebec Artificial Intelligence Institute, Montréal, Canada; 3 Département de Mathématiques et Statistiques, Université de Montréal, Montréal, Canada; 4 Département d’Informatique et Recherche Opérationelle, Université de Montréal, Montréal, Canada; 5 Canada-CIFAR AI Chairs, Montréal, Canada; Research Center Jülich, GERMANY

## Abstract

Neurons in the brain have rich and adaptive input-output properties. Features such as heterogeneous f-I curves and spike frequency adaptation are known to place single neurons in optimal coding regimes when facing changing stimuli. Yet, it is still unclear how brain circuits exploit single-neuron flexibility, and how network-level requirements may have shaped such cellular function. To answer this question, a multi-scaled approach is needed where the computations of single neurons and neural circuits must be considered as a complete system. In this work, we use artificial neural networks to systematically investigate single-neuron input-output adaptive mechanisms, optimized in an end-to-end fashion. Throughout the optimization process, each neuron has the liberty to modify its nonlinear activation function parametrized to mimic f-I curves of biological neurons, either by learning an individual static function or via a learned and shared adaptation mechanism to modify activation functions in real-time during a task. We find that such adaptive networks show much-improved robustness to noise and changes in input statistics. Using tools from dynamical systems theory, we analyze the role of these emergent single-neuron properties and argue that neural diversity and adaptation play an active regularization role, enabling neural circuits to optimally propagate information across time. Finally, we outline similarities between these optimized solutions and known coding strategies found in biological neurons, such as gain scaling and fractional order differentiation/integration.

## Author summary

Evolution has shaped neural circuits in the brain to support complex tasks and behavior. In doing so, single neurons have developed intriguing coding properties such as heterogeneous and adaptive nonlinear responses, and mechanisms at network levels have emerged to efficiently propagate and process information. Discerning the impact of these distinct scales in shaping biophysical mechanisms is a difficult proposition. Are circuits wired the way they are because of single-neuron properties? Are single neurons behaving the way they do because of circuit requirements? To tackle this question, we turn to neural network models that we optimize with deep learning techniques. Crucially, we endow single neurons in these models with parameters that govern their input-output properties, as well as adaptation dynamics. These mechanisms are then optimized end-to-end together with network parameters to perform various perception-like tasks. While this process could have produced any novel solution, we find that what naturally emerges from optimization bears noteworthy similarity to biophysical mechanisms found in neurons in the brain, such as spike-frequency adaptation and gain scaling. We perform an in-depth analysis of the optimization process that uncovered these strategies and formulate hypotheses relating joint evolutionary pressures of single-neuron properties and circuit-level requirements in the brain.

## 1 Introduction

Biological neurons have diverse input responses and adaptation properties [1, 2]. How the rich dynamics of biological neurons combine with network interactions to support complex tasks, such as sensory integration and behavior, remains largely unresolved. While the past decades have seen considerable work aimed at elucidating single neuron coding properties, most efforts have been “bottom up”, modeling mechanistic features observed in biology and analyzing their computational impact. We argue that to shed light on the system-level role of single neuron properties, a “top-down” approach is needed. One way to achieve this is with deep-learning optimization, where “goal-driven” models aim to solve system-level objectives, and emergent neuron properties are studied. In recent years, this method has been extremely successful in capturing single neuron static tuning properties, such as that in the visual system [3]. In this work, we use a goal-driven approach to investigate adaptive input-output properties of neurons that emerge from end-to-end optimization of recurrent neural networks (RNNs), and shed light on their role in biological systems.

A central dynamic component of single-neuron coding is the transformation of input currents into output firing rates neurons execute, as measured by so called *f-I curves*, or *activation functions* (AF). These are both adaptive and diverse across neurons. At the heart of this modularity lies the efficient coding hypothesis, a theoretical paradigm by which neurons aim to be maximally informative about the inputs they encode [4, 5]. Supported by this principle, neurons are known to effectively modulate their f-I curve in response to constant step-like stimulus, in a process know as *spike frequency adaptation* (SFA) [6]. It has been shown that SFA and other adaptive mechanisms in single neurons enable faithful encoding of input signals regardless of stimulus baseline, a crucial feature for animals subject to changing environments [1, 7, 8]. SFA also facilitates information integration over long timescales [9], and provides robustness to rapid variation and noise [10]. At the network level, adaptive neural responses have been shown to support efficient coding with metabolic advantages [11], facilitate computations over long timescales [12–14], and even enable forms of Bayesian inference [15, 16]. Recent work also shows robustness gains from learned modulated neural dynamics [17] and from diverse and dynamic synapses and f-I curves [18, 19], While a number of coding advantages of diverse and dynamic single neuron responses are now established, it is still unknown how these mechanisms have come to bear, and how they influence learning and configuration of larger neural networks that support system-level tasks such as perception or prediction.

In parallel, modern artificial neural networks used in artificial intelligence (AI) loosely mimic neural responses with simple AFs (also called *nonlinearities*) which transform summed inputs to an artificial neuron into a scalar state value, akin to a firing rate. While different shapes of activation functions have been used, and even optimized [20], the prevailing sentiment in AI is that a simple AF such as the *rectified linear unit* (ReLU) is enough for large networks to implement almost any transformation. In fact, this is mathematically guaranteed by the universal function approximation theorem, stating that large enough nonlinear neural networks can implement any function [21, 22]. Reconciling the diverse and dynamic nature of biological neurons’ input-output properties with the computational function of the large networks in the mammalian brain, for example, is therefore a tricky exercise. The prevalent hypothesis is that the single neuron input-output richness found in the brain has evolved and been optimized to guide network-level function such as stable population dynamics, and coordinated learning.

In this work, we propose a step towards complementing longstanding mechanistic investigations into f-I-response diversity and adaptation, through the lens of goal-driven optimization. Using simple artificial neural networks and deep learning, we ask: given the possibility to implement a wide range of single neuron input-output properties, including rapid adaptive mechanisms, do networks optimized end-to-end develop biologically realistic solutions at the single neuron level? If so, can we reconcile single-neuron properties with network-level mechanisms? To address this, we concentrate on the problem of perception on sequential stimuli, such as visual input streams. Our goal is to prescribe the simplest recurrent neural network (RNN) possible that has enough flexibility to develop optimal solutions for its units’ AFs. As such, we propose a two-parameter family of AFs mimicking the diversity of f-I curves that can be implemented by known neural types, and interpolating between often used nonlinearities in AI. In addition, we implement a dynamic controller that modulates AFs in real time, acting locally and independently at each neuron. This controller, implemented with a distinct and smaller RNN, models the genetically encoded adaptation strategy that would have been refined by evolution (see e.g. [17, 23] for similar ideas). We then train this system end-to-end on sequential classification tasks. We call our novel adaptive artificial neuron *Adaptive Recurrent Unit* (ARU).

Our findings can be summarized in three points. First, we find that both diverse and adaptive AFs help RNNs learn tasks, and provide surprising robustness to noise and distractors. Second, we analyze the learned activation functions and adaptation strategies that led to these solutions and find that diversity and adaptation acts as a dynamic regularizer, enabling RNNs to remain in a regime close to the edge of chaos where information transmission and error gradients propagate optimally. Finally, we investigate the learned adaptive AF mechanisms emerging from the optimization procedure by providing a more mechanistic account, comparing them to known biophysical neuronal attributes. We find that optimal AFs take on more biologically plausible configurations (i.e. not simple sigmoid or ReLU), that diversity of AFs is an important and learned feature for robustness, and crucially, that the adaption controller implements a mixture of integrator and differentiator behavior, including leaky integration and fractional order differentiation.

## 2 Results

### 2.1 Flexible activation functions in recurrent neural network models

**Fig 1:**
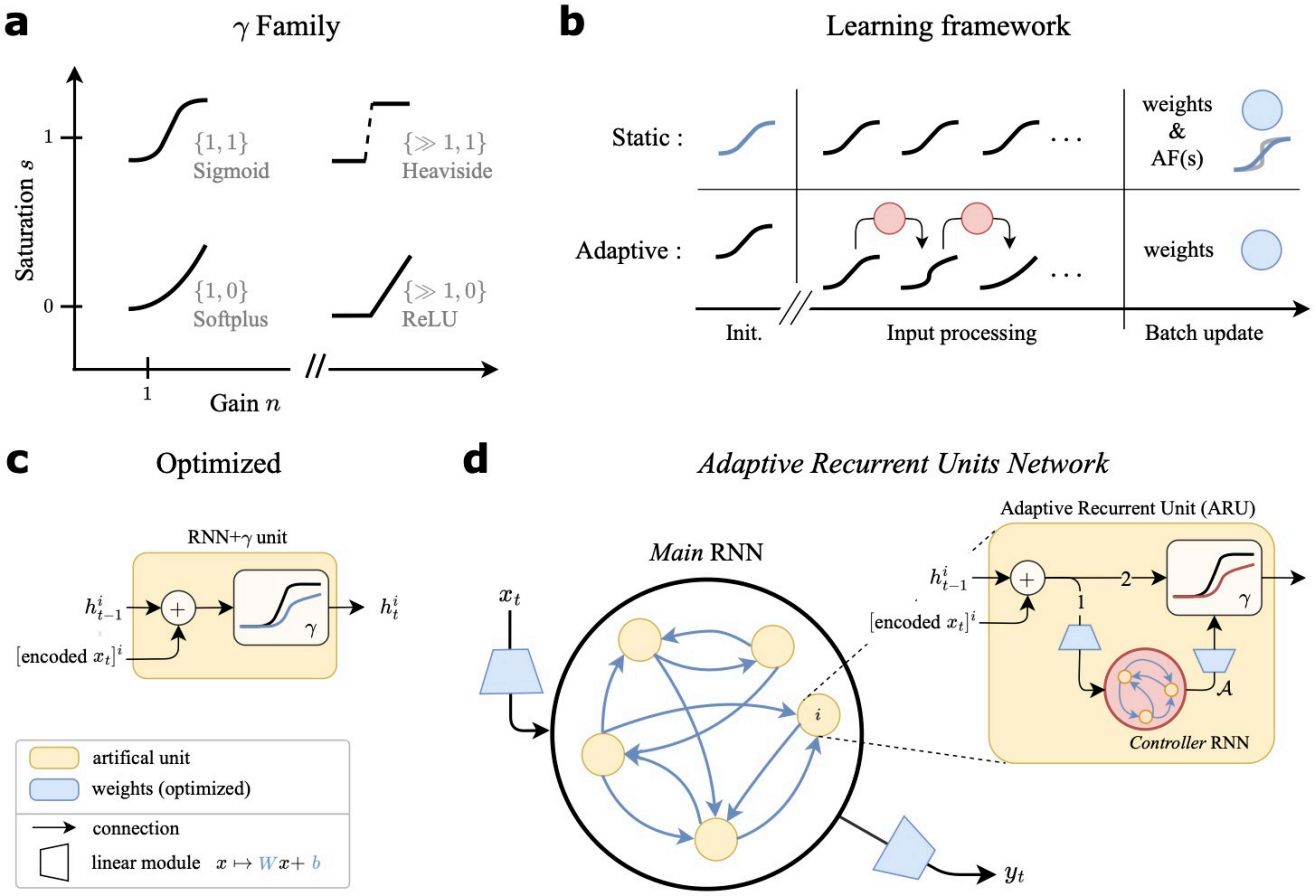
Model details. (**a**) Various shapes of the *γ* activation function (AF), represented in the parameter space {*n*, *s*}. We attain commonly used nonlinearities. (**b**) Different settings considered for modulation of the AF. This modulation is either done alongside training during weight updates and remains static during input processing (*Static*) or online (*Adaptive*). Blue indicates learned parameters; notice the blue activation function in the *Static* setting, updated alongside weights. (**c**) Artificial unit *i* of a standard RNN with *γ* activation function. Legend at the bottom applies to (**c**-**d**). (**d**) Graphical depiction of the Adaptive Recurrent Units (ARU) and associated recurrent network model. Numbers {1, 2} on the arrows in the ARU represent the order of processing. Removing the adaptation mechanism A, we recover the RNN+*γ* model in **c**. Each neuron has a private copy of the controller RNN (in red).

In line with the goal of isolating the role of activation functions, we elect to use a simple “vanilla” RNN model for experiments. The vector equation for the recurrent unit activation $h_{t}\in R^{N_{h}}$ in response to input $x_{t}\in R^{N_{x}}$, *t* ∈ {0, …, *T*} is given by

$$\begin{array}{c} h_{t}=\gamma \left(W_{hh}h_{t-1}+W_{xh}x_{t}+b_{h}\;;n,s\right) \end{array}$$ (1)

where the output $y_{t}\in R^{N_{y}}$ is generated by a linear readout ***y***_*t*_ = *W*_*hy*_***h***_*t*_ + ***b***_*y*_. Weight matrices *W*_(⋅)_ and biases *b*_(⋅)_ are optimized via gradient descent and backpropagation through time (BPTT) in all experiments (see Methods). Departing from standard RNNs, the AF (or nonlinearity) *γ* comes from a differentiable family parametrized by

$$\begin{array}{c} \gamma \left(x;n,s\right)=\left(1-s\right)\frac{\text{log}(1+e^{nx})}{n}+s\frac{e^{nx}}{1+e^{nx}}, \end{array}$$ (2)

for scalar input *x*, with two parameters controlling its shape: the **degree of saturation** *s* and **neuronal gain** *n* (we often shorten these to *saturation* and *gain* and collectively refer to them as the **activation parameters**). This is a *s*-modulated convex sum of two differentiable functions: the non-saturating softplus (*s* = 0), and the saturating sigmoid (*s* = 1), while *n* rescales the domain and controls response sharpness, or gain [24]. Fig 1A shows the graph of *γ* for different values of (*n*, *s*), interpolating between well-known activation functions in deep learning. This also captures key properties of f-I curve shapes present in different neuronal types. For instance, Type I neurons show gradual frequency increase with increasing applied current (i.e. *γ* with low *n*, *s* ≥ 0), whereas Type II neurons show sharp firing onset at non-zero frequencies (i.e. *γ* with high *n*, and low *s* > 0). We let each neuron have their private AF, with activation parameters {*n*^*i*^, *s*^*i*^} associated with neuron *i* (see Section B.1 in S1 Appendix for a comparison to when AF parameters are shared). As *γ* is differentiable in both *s* and *n*, one can include these parameters in the optimization scheme. We consider two main learning frameworks for analysis: (1) *static*, and (2) *adaptive* AFs (see Fig 1B for a schematic). **(1) Static**: each neuron’s AF is optimized by gradient descent (see Methods §4.3) but remains static throughout neural dynamics during input processing (Fig 1C). **(2) Adaptive**: the AF of each neuron *i* has parameters $\left\{n_{t}^{i},s_{t}^{i}\right\}$ varying in time *t*, governed by parameterized mechanisms optimized through gradient descent.

In the adaptive setting, our goal is allow for **adaptation mechanisms** to be discovered by optimization, mapping neural pre-activations $a_{t}^{i}$ to a nonlinear activation function $\gamma (\cdot \;;n_{t}^{i},s_{t}^{i})$, akin to spike-frequency adaptation in cortical neurons. To this end, we train an **adaptation controller** to produce the dynamics of $\left\{n_{t}^{i},s_{t}^{i}\right\}$ (Fig 1D). This controller is itself an RNN module, chosen because of its universal function approximation properties, and does not represents neural circuits like the main RNN. The adaptation controller takes in the pre-activation inputs to a neuron *i*, and returns AF parameters via linear readouts at each time step. Crucially, each neuron has an identical copy of this controller RNN (shared parameters), but each copy controls its single neuron independently of the others. The equations for the controller RNN are given by

$$\begin{array}{c} g_{t}^{\left(i\right)}=\text{tanh}(W_{ag}a_{t}^{i}+W_{gg}g_{t-1}^{\left(i\right)}+b_{g}) \end{array}$$ (3)

$$\begin{array}{c} n_{t}^{i}=W_{gn}g_{t}^{\left(i\right)}+b_{n},\;s_{t}^{i}=W_{gs}g_{t}^{\left(i\right)}+b_{s} \end{array}$$ (4)

$$\begin{array}{c} h_{t}^{i}=\gamma (a_{t}^{i};\;n_{t}^{i},s_{t}^{i}) \end{array}$$ (5)

where $a_{t}^{i}$ is the pre-activation input to neuron *i* at time step *t* (given by $a_{t}^{i}=W_{xh}^{i,:}x_{t}+W_{hh}^{i,:}h_{t-1}+b_{h}$ following Eq 2), and $g_{t}^{\left(i\right)}\in R^{N_{g}}$ is the vector of hidden states for the controller network associated to neuron *i* at time *t*. The controller parameters $\Theta_{A}=\left\{W_{ag},W_{gg},W_{gc},b_{g},b_{c}\right\}$ are shared across all neurons *i* = 1, …, *N*_*h*_, and are optimized end-to-end (see Methods for details). The adaptation controller is thus a shared mechanism across neurons, but with each neuron having a private copy for local control. This is similar to the shared genetic information governing complex ionic machinery, but which individual neurons operate independently from others.

### 2.2 Neural adaptation and diversity improves RNN performance and robustness to input perturbations

We use basic perceptual classification tasks to explore static and adaptive AF optimization in our RNN: (1) sequential classification: MNIST [25] digits from a permuted sequential sequence of pixels (**psMNIST**), and (2) a grayscaled and sequential version of the CIFAR10 classification task (**sCIFAR10**). In both cases, an image is presented to the RNN one pixel at a time, and class membership is inferred at the end (digit number for sMNIST, and “truck”, “plane”, “horse”, etc. for sCIFAR10). See Materials and methods for further details on both tasks. As baseline models for comparison, we use a standard RNN+ReLU [26], as well as the gated LSTM [27] and GRU [28] architectures. The latter two models are known to be more efficient at learning long time-dependencies thanks to architectures that leverage gates. This is, however, less biologically plausible since it relies on network-level mechanisms. Nevertheless, these models offer a good comparison to highlight the advantages of optimized single neurons AF mechanisms, which are closer to biologically plausibibility. As the sCIFAR10 task is more difficult, we also consider a scenario where a convolutional encoder is trained to pre-process inputs. This is optimized end-to-end for each model compared. In all model comparison, we aim to maintain comparable parameter counts (see Materials and methods).

#### Optimized activation functions improve performance

First, we assess model performance by their classification accuracy on held-out test data. We find that the introduction of optimized *γ*(⋅; *n*, *s*) AF, both static and adaptive, provides a considerable increase in performance compared to baselines (see Table 1). On the psMNIST task, RNN+ *γ* (static) outperform both ReLU and gated (LSTM, GRU) baselines. In this testing setting, we did not observe a significant difference between the RNN+*γ* and the adaptive ARUN. Similar results are obtained on the sCIFAR10 task. First using only a linear encoder module like the psMNIST task, we observe that while the GRU offers the highest performance by far, the optimized RNN+ *γ* achieves greater classification accuracy and provides a significant improvement over the RNN+ReLU and LSTM. The introduction of a convolution encoder module significantly increased all the networks’ performance. With this encoding scheme for the sCIFAR10 task, all models offer similar performance.

**Table 1:** Test accuracy on held-out data on the permuted sequential MNIST and sequential CIFAR10 classification tasks. Performance is evaluated on held-out standard input samples (“Original”), and on those same inputs but with a noisy step perturbation ($\xi_{t}∼N(5,2^{2}I$) for psMNIST, $\xi_{t}∼N\left(1,I\right)$ for sCIFAR10) applied to the neurons directly (“Noise Pert.”, see Fig 2a-top). Mean and standard deviation over three random seeds.

(%)	psMNIST		sCIFAR10		
	Original	Noise Pert.	Original		Noise Pert.
Model			Lin. encoder	Conv. encoder	
RNN+ReLU	90.1±0.2	33.0±18.9	28.1±0.2	75.1±2.0	50.0±9.9
LSTM	89.6±2.6	–	36.4±2.3	73.9±1.5	–
GRU	93.3±0.2	–	61.3±0.4	74.2±2.0	–
RNN+*γ* (static)	95.4±0.5	84.0±9.1	42.5±3.1	75.4±1.9	54.2±2.4
ARUN	95.4±0.2	94.7±0.4	44.3±2.9	75.1±2.2	52.3±9.6

#### Adaptive units improve robustness to changes in environment

**Fig 2:**
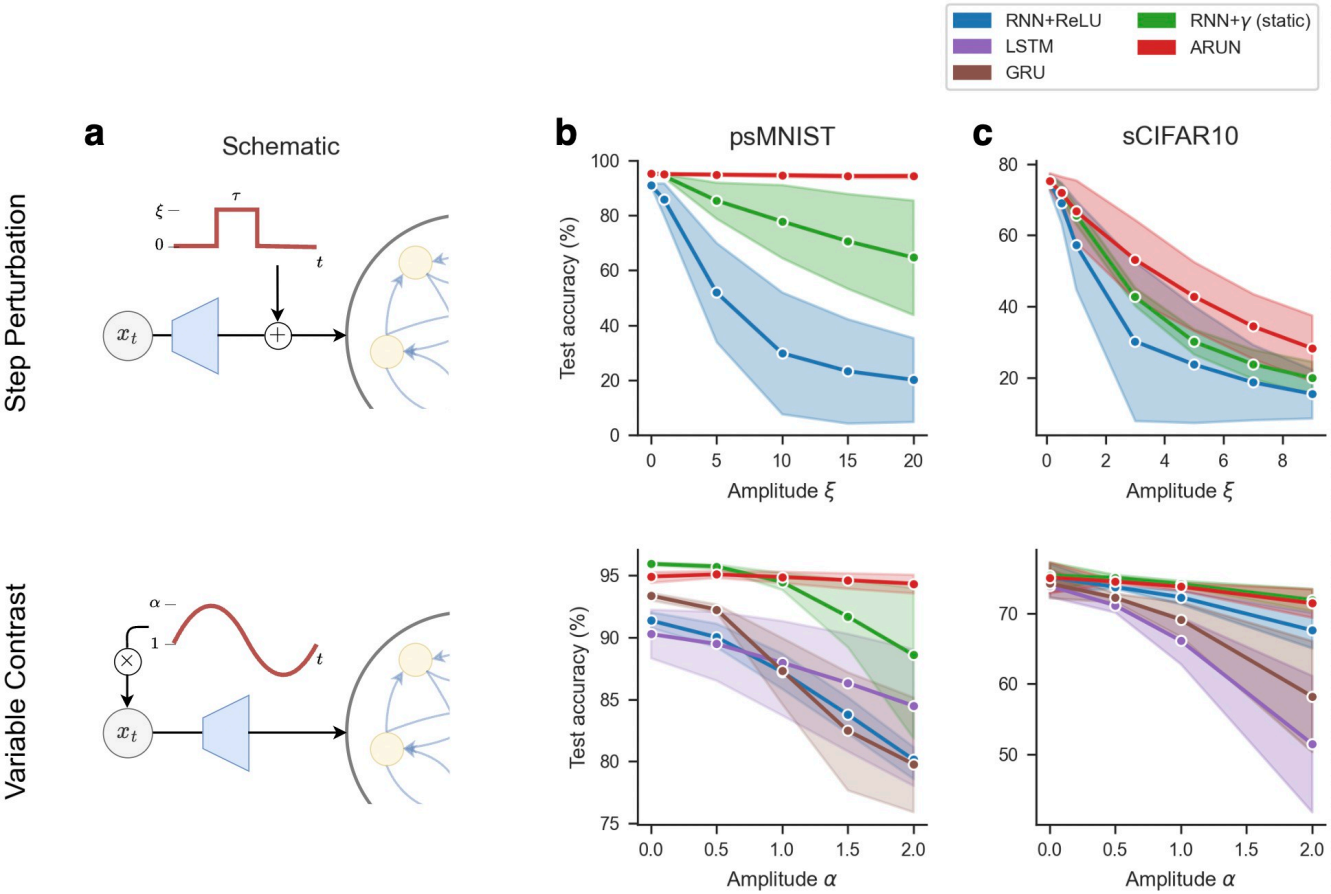
Performance and robustness of RNN architectures on sequential classification tasks. (**a**) Schematic of the perturbations considered, either an additive step perturbation of amplitude *ξ* > 0 for *τ* time-steps (top), or a multiplicative change in contrast applied to the pixels inputs (bottom). Performance as test accuracy on held out data on the permuted sequential MNIST (**b**) and sequential CIFAR10 (**c**) classification tasks. We plot mean trajectories and shading indicates one standard deviation, over three random seeds. For the additive step perturbation analysis (top), we fixed the step perturbation period at *τ* = 200 time-steps starting at *t* = 200, and plot the performance as a function of the drive amplitude *ξ*. Similar qualitative results hold for noisy step perturbations (see Table 1). For the multiplicative variable contrast analysis (bottom), we consider a sinusoidal transform of fixed phase 0, of frequency of 1.0 for psMNIST and 2.0 for sCIFAR10 (best overall performance across networks) and plot the performance as a function of the transform amplitude *α*.

We now study model robustness to perturbations unseen during training. First, we draw inspiration from optogenetic stimulation and inject an external drive *ξ* > 0 directly to each neuron before the AF is applied during *τ* time-steps (Fig 2A-top). Such external drive can either be noisy, drawing $\xi ∼N(\mu_{\xi },\sigma_{\xi }^{2})$ i.i.d. across neurons and in time from normally distributed noise of non-zero mean *μ*_*ξ*_ > 0 and *σ*_*ξ*_ such that *ξ* is almost surely positive, or non-random at a fixed amplitude *ξ* > 0. For those step perturbations we do not consider the LSTM and GRU architectures, as there is no parallel to injecting noise to the main network due to their multiple gating features. Second, we transform the network-level inputs *x*_*t*_ by applying a sinusoidal change in contrast (Fig 2A-bottom), thus altering the input statistics directly. More details in Section 4.5 in Materials and methods.

We observe that networks with adaptive nonlinearities provide an increased ability to mitigate the changes in input statistics from the transformed digits or in response to an added stimulus. In psMNIST, the ARUN outperforms other architectures on the noisy drive (Table 1), and shows high robustness to variable drive amplitude (Fig 2B-top). For varying amplitude on the multiplicative contrast experiment, we observe that the RNN+*γ* model already offers improved robustness, second to again the ARUN showing the lowest decrease in performance. Performance experiments on the sCIFAR10 task are slightly less conclusive, as expected, attributable in part to the overall lower performance for all networks. We nonetheless observe that the ARUN still offers more robustness to step perturbations (Fig 2C-top), and overall lessend decrease in performance on the variable contrast (Fig 2C-top) with respect to baselines.

On top of the results presented in Fig 2, we conducted a sensitivity analysis with respect to the various parameters of the transformations (see Fig I and Fig L in S1 Appendix). First, we varied the phase and amplitude of the sinusoidal transformation applied on inputs, and we observe that the ARUN presents the best robustness on the psMNIST task. Second, we varied the amplitude and length of the step-drive applied on neurons. In this driven case, again on the psMNIST task, the ARUN presents a test loss of an order of magnitude lower than the other RNN models while varying the parameters. Similar sensitivity analysis on sCIFAR10 suffers from a similar lack of significant differences between models as the results presented previously. We still observe similar trends towards the beneficial impact of our adaptive architecture. Thus in all, endowing networks with adaptive nonlinearities presents advantages in mitigating changes in input statistics.

### 2.3 Neural adaptation improves global network information propagation

So far, we have established that introducing the freedom to learn diverse and/or adaptive tuning of single neuron AFs improves an RNN’s performance on perceptual tasks, and enables enhanced robustness to noise. In this section, we further analyze the adaptive controller’s dynamics to uncover what are the ARU mechanisms that emerge from end-to-end optimization, how they support robustness.

**Fig 3:**
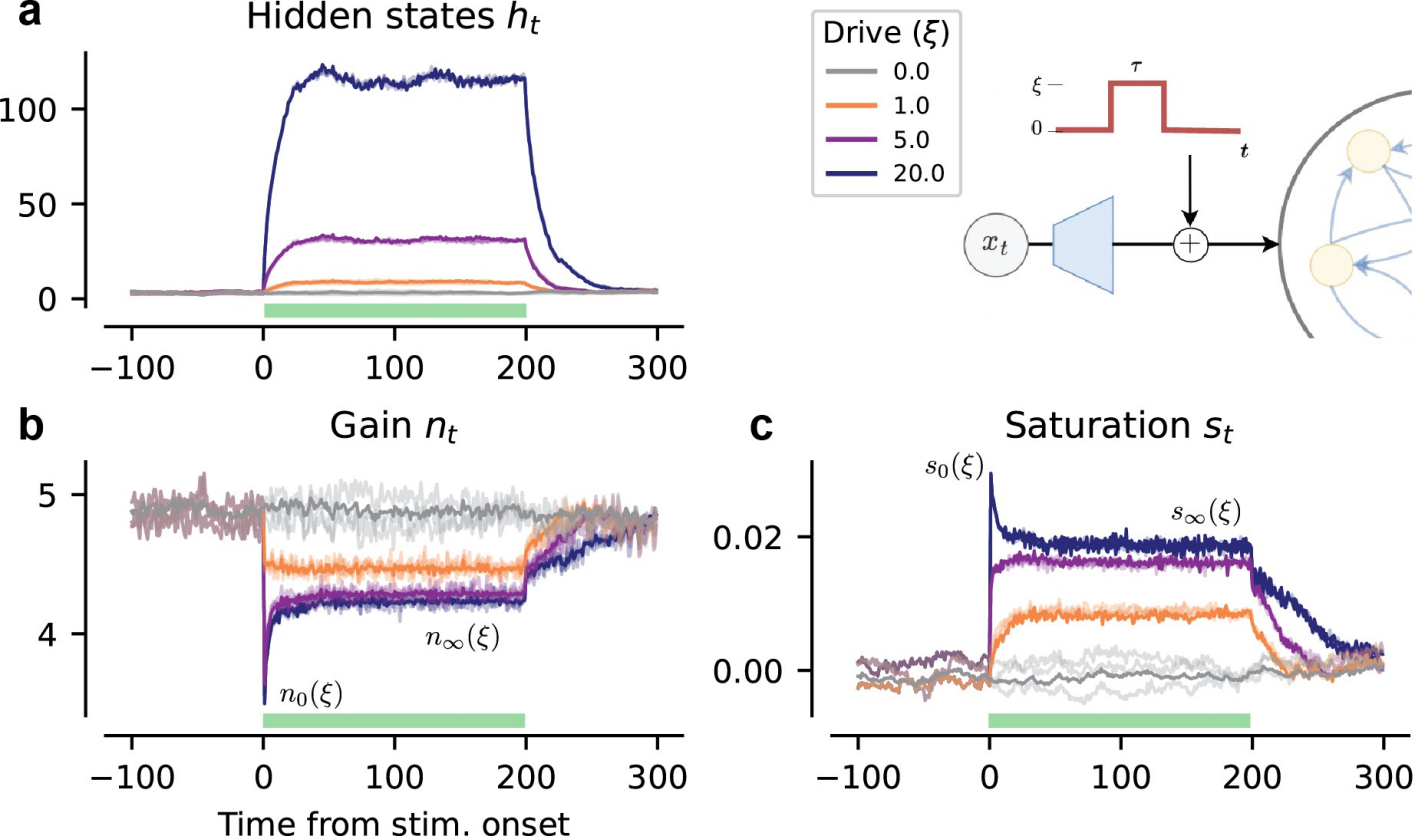
ARU responses to a step perturbation. Population-averaged hidden states *h*_*t*_ (**a**), gain *n*_*t*_ (**b**) and saturation *s*_*t*_ (**c**) as a response to a step pertubation with varying amplitude *ξ* (*ξ* legend reported right of **a**), with schematic of step perturbation as in Fig 2. Step-perturbation duration is indicated by the shaded green region, with time steps aligned with perturbation onset. We indicate the onset {*n*_0_(*ξ*), *s*_0_(*ξ*)} and steady-state {*n*_∞_(*ξ*), *s*_∞_(*ξ*)} activation parameters. Light shaded lines represent 3 exemplar single-neuron traces.

**Fig 4:**
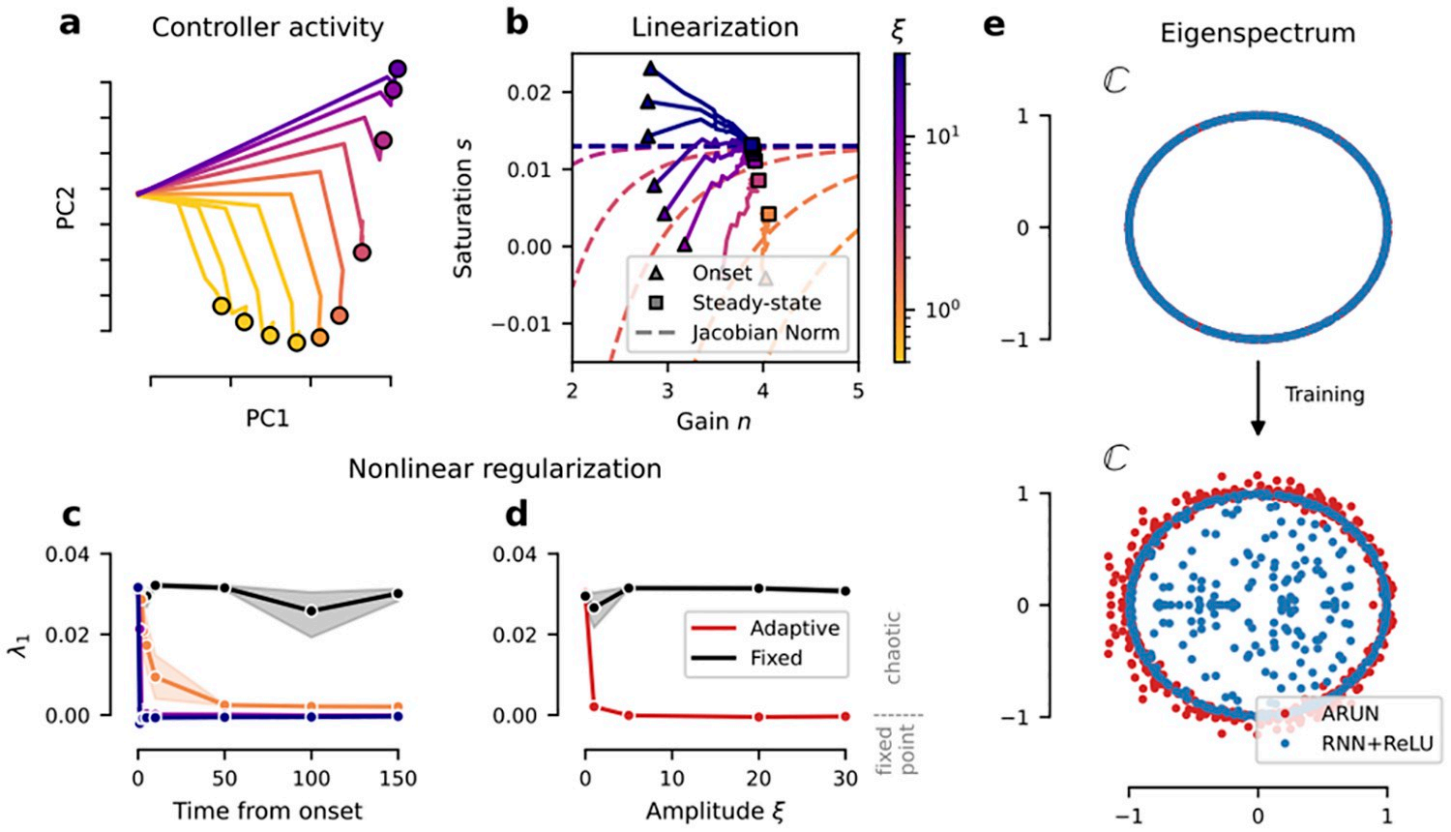
Dynamic regularization by ARU controllers. **(a)** Limit points in controller activity from constant input *ξ*. **(b)** {*n*_*t*_, *s*_*t*_} trajectories during stimulation, with onset (triangle) and steady state (square), for each external drive *ξ*. Overlayed over jacobian *γ*′(*ξ*;*n*, *s*) = 1 − *ϵ* level curves in phase space (*ϵ* = 0.01). Colorbar shared with (**a**-**c**). **(c-d)** Maximum Lyapunov Exponent (*λ*_1_) of an ARUN’s *main* RNN averaged over *n* = 10 draws from the stationary distribution (mean and 95% c.i. shown, details in Section 4.4 in Materials and methods). The black line indicates the original *ξ* = 0 setting. (**c**) *λ*_1_ as a function of the chosen time-step $\hat{t}$ from the onset of the perturbation, for varying *ξ* (see colorbar in **b**). We take $\left\{n_{\hat{t}}\left(\xi \right),s_{\hat{t}}\left(\xi \right)\right\}$ set by an ARU and treat them as unvarying in time (equivalent to RNN+*γ* static). (**d**) *λ*_1_ as a function of the step-drive amplitude *ξ*. We take the steady-state values {*n*_∞_(*ξ*), *s*_∞_(*ξ*)} (*ξ* > 0 for “Adaptive”, *ξ* = 0 for “Fixed”). (**e**) Eigenvalues of the (main) RNN recurrent connectivity matrix *W*_*hh*_ before (top) and after (bottom) training. The eigenvalues have an average norm 1.02 (with quartiles [Q1, Q3] = [0.98, 1.05]) for the ARUN and 0.83 (with quartiles [Q1, Q3] = [0.69, 1.00]) for the RNN+ReLU.

We begin by focusing on the {*n*_*t*_, *s*_*t*_} trajectory, governing the AF of a single neuron when presented with a step-input—see Fig 3 for ARU activity trajectories for different perturbation amplitudes *ξ*. At stimulus onset, we observe an initial spike with *ξ*-dependent onset values we name {*n*_0_(*ξ*), *s*_0_(*ξ*)}. Following this is an exponential decay toward *ξ*-dependent steady state values {*n*_∞_(*ξ*), *s*_∞_(*ξ*)}. This transient decay is responsible for differentiation and integration, while the values {*n*_∞_(*ξ*), *s*_∞_(*ξ*)} dictate the magnitude of steady-state gain scaling, both of which we investigate further in in Section 2.4. Fig 4A shows the dynamics of the controller RNN whose output is the {*n*_*t*_, *s*_*t*_} trajectory, projected in the first two principal components (PCs) of the controller’s hidden states (*N* = 50, two PCs account for 96% of explained variance). Markers indicate steady states of the controller under constant input *ξ* following the transient decay, and lead to outputs {*n*_∞_(*ξ*), *s*_∞_(*ξ*)}. These clearly lie on a seemingly continuous 1-D manifold which encodes the amount of scaling the neuron’s AF receives under prolonged constant input. In what follows, we aim to uncover what coding advantages these adaptive dynamics afford, first at the single neuron level, then for networks.

#### Adaptive activation reduces noise variance in single neurons

We now quantify the impact of the activation function *γ* and its parameters {*n*, *s*} on noise integration in single neurons. As a response to a general perturbation scalar $\eta ∼N(\mu ,\sigma^{2})$, we seek to quantify the role of the parameters {*n*, *s*} in amplifying or reducing this noise. To this end, we present the following proposition.

**Proposition 1**. *For unitary W*_*hh*_ *weight initialization, the variance explained along a vector* $u\in R^{N_{h}}$ *as a response to a perturbation* $\eta ∼N(\mu ,\sigma^{2}I)$ *decays if and only if the parameters* {***n***_*t*_, ***s***_*t*_} *satisfy*

$$\begin{array}{c} \sigma^{2}[\frac{d}{dx}\gamma (x\;;n_{t}^{i},s_{t}^{i}){|}_{x=\mu^{i}}{]}^{2}<1+O(\sigma^{3}) \end{array}$$ (6)

*for i* ∈ {1, …, *N*_*h*_}.

*Proof*. See proof in Section C.2 in S1 Appendix.

This result leverages known conditions on an RNN’s Jacobian under mild connectivity assumptions to ensure noise inputs are not amplified. For example, for a linear AF with slope *a*, we would require *σ* ≤ 1/*a* to avoid noise amplification. For a neuron with AF *γ*(*x*; *n*, *s*) and small *ϵ* > 0, the continuity of *γ* and the implicit function theorem guarantees that there exists a 1-D manifold in {*n*, *s*}-parameter space indicating a stability boundary. We can derive this manifold parametrized by the drive’s amplitude *ξ* by solving for $\sigma^{2}[\frac{d}{d\xi }\gamma (\xi \;;n_{t},s_{t}){]}^{2}=1-\epsilon$. This path corresponds to the expected variation in activation parameters {*n*, *s*} as a function of *ξ* for the system to absorb, through the hidden-state dynamics, the injected noise by a margin *ϵ*. This result requires linearization assumptions, see Section C in S1 Appendix for further details.

Equipped with this result, we return to the ARU adaptive dynamics obtained by optimization. Fig 4B shows the activation parameter trajectories plotted in the {*n*, *s*}-plane. Triangle markers indicate onset values {*n*_0_, *s*_0_} in response to step inputs, while square ones indicate steady states {*n*_∞_, *s*_∞_}. Dashed lines indicate the manifolds predicted by the above results, where color indicates corresponding input amplitude *ξ*. We can observe that for moderate to high *ξ*, our theory accurately predicts end states of {*n*_*t*_, *s*_*t*_} dynamics. The prediction degrades for low input amplitudes, a regime for which the main network can compensate noise by itself without neural adaptation (see Fig 2). Overall, this indicates that in the presence of large perturbations (unseen in training), the adaptation-modulated gain-scaling operation described in Section 2.4 places neurons in a stable dynamic regime that avoids noise amplification.

#### Adaptation maintains network dynamics at the edge of chaos

Beyond single neuron mechanisms, we now turn to network-wide effects of adaptive units, and ask how adaptive units influence population dynamics. To better quantify this, we leverage Lyapunov Exponents, a measurement of average expansion and contraction of state space by a dynamical system. The sign of the largest exponent (*λ*_1_) is a good indicator of a system’s stability: chaotic systems have at least one positive exponent. A system with *λ*_1_ = 0 is said to be at the “edge of chaos”, with computational advantages we discuss in detail in the Discussion section. We approximate *λ*_1_ for RNNs as described in [29] with, and without adaptation. We refer to Section 4.4 in Materials and methods for details on the computation, and Section D in S1 Appendix for details and a primer on the topic.

We find that for constant drives of distinct amplitude *ξ* > 0, adaptation mechanism actively steer the networks toward λ_1_ several orders of magnitude closer to 0 in comparison to non-adaptive RNNs (see Fig 4C and 4D). This means that in situations where inputs are strong enough to destabilize dynamics in a non-adaptive RNN (thus leading to loss in performance), networks with ARUs remain more stable at the edge of chaos.

This mechanism is further showcased when observing the eigenspectra of optimized RNN connectivity matrices. In a standard non-adaptive RNN, connectivity matrices with eigenvalues whose magnitude are greater than one typically lead to chaotic dynamics, while smaller eigenvalues indicate stable dynamics. If RNNs were linear, the log eigenvalue norms directly indicate Lyapunov exponents. Therefore, it is widely known that initializing RNNs with unit norm eigenvalues leads to dynamics at the edge of chaos. One such initialization strategy is to initialize at the identity, or to select orthogonal matrices, also known as the Henaff initialisation [30]. In Fig 4E, we compare optimized connectivity matrix eigenspectra of adaptive and non-adaptive RNNs with the same Henaff initialization and training procedure. What we observe is that adaptive RNNs eigenspectra have a magnitude well beyond the unit circle, in contrast to non-adaptive RNNs. Since we know from Lyapunov exponents that adaptive RNNs operate at the edge of chaos despite this connectivity, this indicates a form of “dynamic damping” provided by adaptation. As further explored in the discussion below, this suggests that adaptation at the single neuron level enables added expressivity by allowing a wider range of recurrent connectivity, while maintaining stability across dynamic regimes.

### 2.4 Top-down optimization of adaptive RNNs reveals rich single neuron coding mechanisms

**Fig 5:**
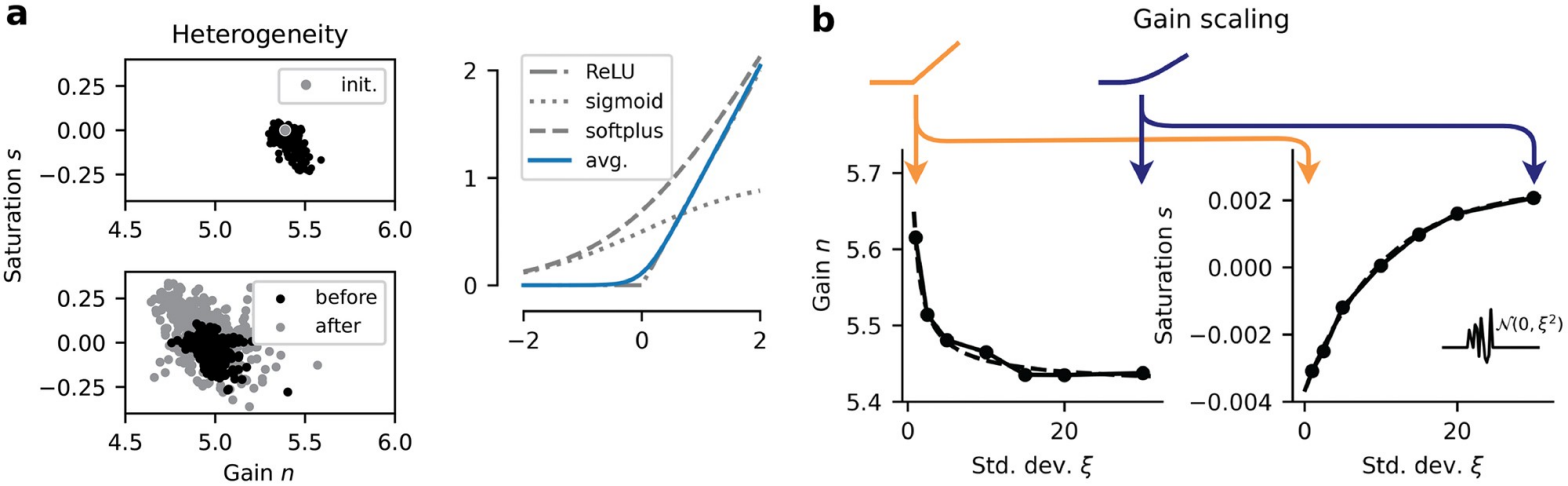
Steady-state coding mechanisms of ARUs. (**a**) Neuron-to-neuron heterogeneity in AFs. (Top) Learned activation parameters in RNN+*γ* (het.), from homogeneous initialization. (Bottom) slight increase in heterogeneity before and after a 45° rotation is applied to the psMNIST digit, learned through backpropagation. (Right) The learned activation function shape corresponding to the average {*n*, *s*} parameters on the left (Top), shown along common AFs part of the *γ* AF family. (**b**) As a response to a noisy external drive of varying variance, the gain of the ARUs displays power-like decay (fit in dashed) as a function of the std.-dev. *ξ*, and the saturation displays exponential ramp-up (fit in dashed). Averaged over *N* = 3 seeds. Depictions of the associated *γ* AFs are included above, exaggerated for visualization purposes (color follows colorbar).

**Fig 6:**
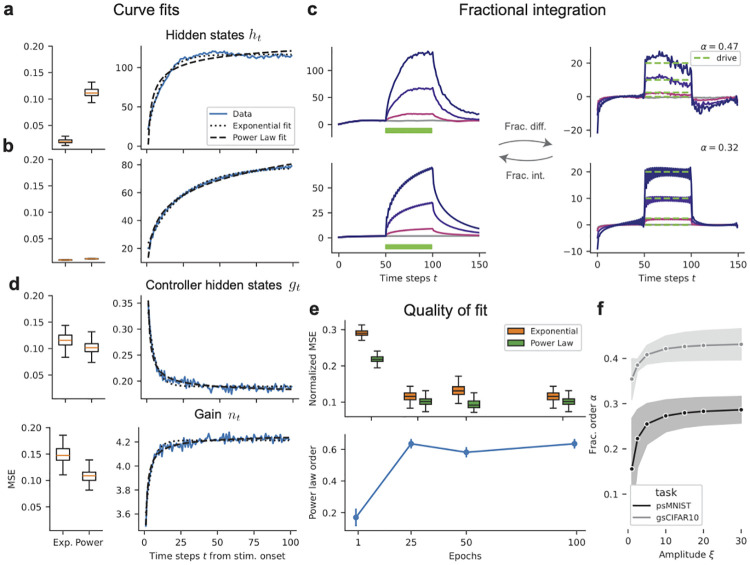
Differentiation and integration behavior by ARUs. **(a, b, d)** Curve fit results, with the standardized MSE on the left and example curve fits to population averages on the right, for the ARU standard (**a**) and non-interacting (**b**) hidden states *h*_*t*_, as well as in (**d**) the controller hidden states *g*_*t*_ and the gains *n*_*t*_, during the step perturbation. Box-plots correspond to MSEs over *B* = 100 different independent input sequences, and we use the average fit parameters on the right. (**c**) Standard (top) and non-interacting (bottom) hidden states, before (left) and after (right) applying the fractional order differentiation. The original provided step perturbation “drive” is plotted against the fractional-order differentiated signals, whose order is indicated in the top right. (**e**) Curve fits to the population-averaged *g*_*t*_ over training epochs, with the quality of fit (top) and the power law order *α* (bottom). (**f**) Fractional order, per task, for varying step-perturbation amplitude *ξ*. Shaded regions indicate one standard deviation around the mean, over 3 random seeds.

When trained on temporal perception tasks (sequential MNIST/CIFAR10), we demonstrated in Section 2.2 that our network of ARUs shows improved robustness to noise and changes in input statistics. Furthermore, as highlighted in Section 2.3, while our adaptation mechanism is local and independent across neurons, the combined effects actively balance global network dynamics in response to changes in input statistics and conserve stable computations. In this section, we provide a more observational perspective and compare the emergent adaptation mechanisms to biophysical neuronal mechanisms. Specifically, we focus on: (1) steady-state mechanisms, such as heterogeneity and gain scaling (Fig 5) and (2) online adaptive mechanisms (Fig 6), through the lens of fractional differentiation and integration. We stress that our RNNs with adaptive capabilities have minimal inductive biases and that optimization could have, in principle, led to any AF adaptive mechanisms regardless of biological realism.

#### Steady-state mechanisms

We demonstrated in Section 2.2 that single-neuron activation flexibility, either static or adaptive, was beneficial to task performance and robustness. In static networks, we observe that when the activation function *γ* is initialized identically for every neuron, the optimization procedure leads to moderate heterogeneity in the AF parameters across neurons in the network (Fig 5A-top). Ref. [19] shows similar results when AFs are parametrized following known relations between ionic currents and f-I curves. This flexibility at single-neuron level did moderately increase performance (see Section B.1 in S1 Appendix). We note that this learned heterogeneity (Fig 5A-top) is relatively minimal, with most learned activation functions still resembling the soft-plus function (see average in Fig 5A-right). Further experiments (details included in Section B.3 in S1 Appendix) consider trained RNN+*γ* networks reading psMNIST digits rotated by 45°. As opposed to the perturbation experiments highlighted previously, this also changes the temporal order in which the inputs are fed. In this new setting, we observed an increase in {*n*, *s*} heterogeneity upon changes in task temporal statistics (Fig 5A-bottom), when all other parameters are kept fixed. Simply allowing the AFs to be modulated could recover over a quarter of lost performance (over 25%) in this altered task. In sum, these results support that single-neuron activation function diversity is learned through optimization, and is beneficial to performance and robustness.

In Section 2.3, we investigated the role the mean steady-state response value of the activation parameters {*n*_∞_(*ξ*), *s*_∞_(*ξ*)} plays on the stability of network dynamics. We find this gain adaptation behavior to follow general gain scaling principles of visual sensory system neurons [5, 7]. Indeed, we subjected ARUs to a low-noise signal $N(0,0.01^{2})$ for *t* = 200 time steps, followed by i.i.d samples from $N(0,\xi^{2})$ for varying *ξ* > 0 during another *t* = 200 time steps, before returning to the original low noise (see inlet Fig 5b). We observe the steady-state gain *n*_∞_(*ξ*) to display a decreasing power-law dependence on *ξ* (Fig 5b-left). As for the saturation, we observed a exponential dependence on *ξ* (Fig 5b-right). This type of gain scaling is similar to that of fast-spiking sensorimotor interneurons [31].

#### ARUs as integrators/differentiators

Finally, we study the dynamic coding mechanisms implemented online by ARUs (Fig 3) from the perspective of neural integrator/differentiator behavior. Differentiation behavior can take many forms, from leaky integration [32, 33] to fractional order differentiation. Fractional order differentiation is a fundamental mechanism for efficient information processing in cortical neurons intimately related to gain scaling [2, 10, 34]. It is a convolution in time domain, defined by the product in Fourier domain of the transformed signal with the operator *H*(*f*) = (2*iπf*)^*α*^. By varying the fractional order parameter *α* ∈ [−1, 1], one continuously interpolates between differentiation (*α* = 1) and integration (*α* = −1) of a signal. Both positive and negative orders are mathematically justified, observed in retinal neural populations [35, 36], and carry meaning as a convolutional filter mechanism. Of interest for us, for a stimulus of the form *ξ* ⋅ *H*(*t*) as studied in Section 2.3 where *ξ* ≥ 0 is the step size and *H*(*t*) is the Heaviside step function, a fractional differentiation (resp. integration) response would be proportional to *ξ* ⋅ *t*^−*α*^ for *α* > 0 (resp. *α* < 0).

To determine the type of integration behavior, we analyze the various ARU response signals by fitting exponential (*a* exp(−*t*/*b*) + *c*) and power law (*at*^−*b*^ + *c*) functions to the population-averaged signals during a step perturbation. We evaluate goodness of fit by the mean squared error (MSE) between the curve fit and the signal. We standardize all signals by subtracting off their mean and dividing by the standard deviation—thus as a baseline, the MSE for a fit by a constant function (i.e. the mean) would be 1.0.

From the MSEs (see Fig 6a) we see that the ARU hidden states *h*_*t*_ are noisy and that both exponential and power law offer decent but not perfect fits, with exponential better, making the behavior of ARUs closer to leaky integration. In a setting conceptually closer to the original experiment from [10], performed on slices of neocortical pyramidal neurons, we also consider the same step-constant drives applied to single ARUs in isolation, with only trained self-connection weights. In this “isolated” setting we see that now both the power law and exponential provide a good fit (see Fig 6b), providing evidence for behavior similar to leaky- and fractional integration. Fractional differentiation of the non-interacting hidden states in fact recovers the original step perturbation applied (Fig 6c). Furthermore, we identified single-neuron level heterogeneity in the specific fractional order used. Indeed, while the controller has shared weights across neurons, the unit-specific history dependence makes the adaptive AF process unit-specific. We report in Fig H in S1 Appendix the distribution of fractional order over single-neurons—an investigation of the precise source of this diversity is left to future work. Finally, taking a closer look at the fractional orders obtained from fractional differentiation (see Section 4.4 in Materials and methods), we observe in Fig 6f that they depend on the task considered and the fractional order *ξ*, increasing for low *ξ*-values to a plateau for higher values.

While the fits and the dependency of the fractional order *α* on *ξ* further shows that the ARU hidden states *h*_*t*_ do not exhibit strictly fractional differentiator behavior, we still observe fractional integration characteristics. Now, unlike the main network activity, the controller hidden states *g*_*t*_ (and similarly, the gain dynamics *n*_*t*_ linearly decoded from *g*_*t*_) are better fit by a power law (Fig 6d). This indicates that the ARU hidden dynamics of the *controller* itself are better described as a fractional differentiator. Furthermore, we observe that the quality of fit of the power law to the controller hidden states increases across training (see Fig 6e-top). The order of this power law increases (see Fig 6e-bottom), making the controller have a higher and higher order of fractional differentiation, stabilizing around an order of 0.7. In sum, these results suggest that ARUs implement differentiator-like mechanisms that bear similarity to a combination of known biophysical mechanisms. We reiterate that nothing constrained the network to learn such solutions, and that the observed mechanisms emerged from end-to-end optimization.

## 3 Discussion

**Optimal information propagation through local regularization**. Recurrent neural networks, whether biological or artificial, must balance *expressivity* and *stability* to implement complex computations. A network whose dynamics quickly converge to a single fixed point, for example, is quite stable but not expressive since very little mathematical transformations of inputs can take place (e.g. integration, amplification). In contrast, a network operating in a chaotic regime is expressive but unstable: its dynamics are rich but tiny perturbations lead to widely contrasting outcomes. The balance between these two requirements has been identified in several contexts, and is often referred to as the “edge of chaos” [37]. Dynamics close to the transition point between stable and chaotic dynamics offer optimal information propagation over long timescales, as well as rich transformations [37–39]. This regime has also been shown to be important in deep and recurrent artificial neural networks [40]. Indeed, a rich theory of how large networks learn and implement computations shows that expressivity is maximized when dynamics are close to chaotic.

In machine learning, several strategies have been developed to ensure efficient training of artificial neural networks. Much of these strategies rely on global knowledge of networks [41], and global interventions on connectivity [30, 42–45]. For instance during training, batch normalization has proven surprisingly efficient at enforcing certain dynamical regimes. Gating units such as those found in LSTMs and GRUs have also been shown to help stabilize dynamics (see [46] for a review of the role of multiplicative gating). However, these processes are not biologically plausible as they are inherently non-local, requiring knowledge across space and/or time. While useful in AI/ML, these mechanisms offer little insight into how the brain might maintain dynamics at the edge of chaos under a variety of input statistics and distributed changes in connectivity throughout learning. Our results suggest that diverse and adaptive single neuron properties likely play such a role. Indeed, we demonstrate that ARUs offer a form of biologically plausible dynamic regularization, dampening the system in the presence of changing input statistics in such a way to promote network-level optimality. This contributes to understanding network-level effects of single-neuron adaptation [33]. In that vein, diluting the controller dynamics to be compared to rate-based models of adaptation provides rich grounds for future work. In our experiments, these mechanisms emerge from end-to-end optimization and bear similarity to well-studied biological neuron properties, suggesting adaptive neural dynamics could have evolved to maintain networks in optimal states.

**Deep learning and backpropagation as a framework to uncover biologically realistic optimal solutions**. In this study, we train network models using gradient descent via backpropagation (through time, BPTT) of errors [47], an algorithm that computes error gradients at once for all network parameters (including adaptation controllers). It is unlikely that the brain makes use of backpropagation exactly as it is implemented for artificial network optimization, thanks to the non-locality of credit assignment arising when computing gradients. Importantly, we reiterate that our experiments do not model biological learning, but rather leverages optimality of learned solutions to study emergent mechanisms. Nevertheless, an important question is to ask if optimal solutions found by backpropagation have any reasonable correspondence to those found by evolution and biological learning. We argue that the requirement of stable and expressive information propagation in time is a central commonality shared by optimized artificial and biological networks.

When training RNNs with BPTT, a central challenge is the vanishing and exploding gradient problem [41, 48]. Here, gradient norms tend to either grow or vanish exponentially with time steps, leading to problems when conducting gradient descent. For an RNN whose dynamics are given by *h*_*t*+1_ = *F*(*h*_*t*_, *x*_*t*_), the main culprit for exponential growth and decay when computing error gradients comes from a term involving long products of Jacobian matrices evaluated along trajectories:

$$\begin{array}{c} P_{T}=\prod_{t=0}^{T}D_{h}F\left(h_{t},x_{t}\right). \end{array}$$ (7)

For RNNs following Eq (1), *D*_*h*_*F*(*h*, *x*) = diag(*γ*′(*W*_*hh*_*h* + *W*_*xh*_*x*; *n*, *s*))^⊤^*W*_*hh*_. Given idealized nonlinearity *γ* (e.g. ReLU) it should be clear that requiring eigenvalues of *W*_*hh*_ to be close to unit norm mitigates vanishing or explosion of *P*_*T*_. Crucially, we note that Eq (7) is precisely the variational equation of a dynamical system (see [29]), and that Lyapunov exponents are given by the time-averaged logarithm of spectral quantities of *P*_*T*_ when *T* → ∞.

Therefore, the stability of backward gradient propagation and of forward information propagation are two sides of the same coin. Forward dynamics at the edge of chaos, with Lyapunov exponents close to zero, correspond exactly to a regime where gradient norms avoid exponential growth or decay. In other words, a network operating at the edge of chaos forward in time also propagates error gradients backward in time efficiently. Thus, networks optimized via BPTT, which naturally promotes stable gradient propagation [47], will be pushed to operate at the edge of chaos in forward time. If we assume the brain evolved to operate at the edge of chaos thanks to selective preference for neural circuits that propagate information over long timescales (a prevailing possibility with several experimental confounds, see e.g. [37–39]), then there should be an important overlap between biological and artificial solutions for dynamic stability. As such, despite BPTT not being biologically plausible as a learning mechanism, we argue that ingredients contributing to stable information propagation that emerge from BPTT, such as adaptive mechanisms, are likely consistent with brain evolutionary pressures toward stable and expressive information propagation. We refer the reader to Section C.5 in S1 Appendix for a detailed analysis of gradient propagation in our model. There, we compare gradient norms as they propagate throughout training and found that adaptive networks maintain stable gradient norm better than non-adaptive RNNs trained in the exact same way.

Finally, we note that we did not focus on the issue of multiple optimization timescales. In a more strict comparison to neuron dynamics and neural circuits in the brain, ARU controllers would have been optimized over evolutionary timescales, while the main RNN parameters, representing synaptic connections, over the lifespan of an animal. We did try a limited number of experiments, for example by fixing one while learning the other and vice versa (see Section C.4 in S1 Appendix), and did not see any significant differences in results. A different methodology, borrowing from deep learning frameworks like meta-learning, could allow for a more adequate consideration of adaptive mechanisms as a product of evolution-like pressures (see e.g. [23]). Such a more thorough investigation of the impact of learning timescales on solutions is outside of the scope of this paper, but is a fascinating direction of future work to disentangle evolution and learning pressures.

**Conclusion**. In this work, we sought to investigate goal-driven learning pressures from the system-level onto dynamic coding mechanisms at the single-neuron level. We do so by introducing *adaptive recurrent units*, allowing for online AF control from a novel parametric family. Our main findings are threefold: (1) Diverse and adaptive activation functions improve computational performance of networks while also helping to mitigate previously unobserved changes in input statistics during a task, thus improving out-of-distribution generalization. (2) We find that adaptation acts as a dynamic regularizer allowing recurrent neural networks to remain in a dynamic regime closer to the edge of chaos where forward and backward information propagation is optimal. (3) Finally, system-level learning pressures drive biologically plausible adaptation strategies, namely activation function having biologically realistic configurations and more importantly, the implementation of biological SFA mechanisms such as gain scaling and input differentiation. These findings are supported by detailed numerical experiments and analytically derived bounds for information propagation in networks. We discuss how ARU adaptation can effectively implement a number of methods often used in deep learning to ensure good network expressivity and stability, including regularization and normalization. In contrast to these methods which require global, biologically unrealistic network quantities, ARU adaptation is local to each neuron and is consistent with known physiology. Taken together, our results support that neural diversity and adaptation serves a crucial role in goal-oriented network optimization, which suggests a coordinated and consistent optimality across scales linking brain circuits and single neurons.

## 4 Materials and methods

### 4.1 Tasks

#### psMNIST

(Permuted Sequential MNIST). This task, first proposed by [43], focuses on learning to classify hand-written digits from the MNIST dataset [49] by presenting the pixels sequentially. This requires an accumulation of information over long timescales. Furthermore, we apply a fixed random permutation to the pixels; this reduces the time-step to time-step correlation and thus makes the task harder.

#### sCIFAR10

(Sequential CIFAR10). This second task is fundamentally similar to the previous MNIST one, where here the goal is to classify CIFAR10 [50] images. The network is shown (grayscaled) images of real world objects one pixel at the time and has to determine to which one of the 10 classes the image belongs. Now because the images are of objects in a variety of settings and not simply of digits, this constitutes a significantly harder task than psMNIST.

### 4.2 Network architecture details

For the RNN+*γ* and ARUN networks, we use a network size of *N*_*h*_ = 400 hidden-states. In the ARUN, the adaptation controller is chosen to operate with a smaller network, that we set to *N*_*g*_ = 50. With respect to a standard RNN, like the RNN+ ReLU model, a heterogeneously optimized AF introduces 2 ⋅ *N*_*h*_ new parameters, and an adaptive controller RNN introduces (*N*_*g*_)^2^ + 2 ⋅ *N*_*g*_ parameters. We control the number of hidden-states in the other LSTM and GRU architectures to provide a similar (within 2%) number of parameters across all architectures.

We use linear encoder networks for the psMNIST task. For the CIFAR10 task, we resort to convolutional neural network (CNN) encoding schemes. More precisely we use a CNN with 4 convolutional layers and max pooling after the 2^nd^ and 4^th^ conv. layers. All convolutional layers are directly preceded by batch normalization and an element-wise application of the ReLU activation function and all layers have 3 × 3 sized kernels as well as padding and stride of 1 in both directions. The number of channels in each convolutional layer is 32, 64, 128 and 256 in order from input to output. In both cases max pooling is used over 2 × 2 sized regions with a stride of 2.

### 4.3 Modeling and training details

The vector of all trainable parameters is denoted Θ, and the parameters are updated via gradient descent using backpropagation through time (BPTT), with the matrix *W*_*rec*_ initialized using a random orthogonal scheme [30]. Independently of the task, we used Cross-entropy loss as our loss function and the Adam [51] optimizer. We experimented with the RMSprop optimizer (introduced by [52], first used by [53]) with smoothing constant *α* = 0.99 and no weight decay, which yielded similar results. We trained the networks for 100 epochs. We investigated different learning rates (LR ∈ {10^−3^, 10^−4^, 10^−5^, 10^−6^}), and settled on 10^−4^.

#### Activation parameters

We first consider an initialization grid *N* × *S*, where *N* = {1.0} ∪ {1.25*k*: 1 ≤ *k* ≤ 16} and *S* = {0.0, 0.25, 0.5, 0.75, 1.0} such that |*N*| = 17 and |*S*| = 5. For each pair of parameters $\left\{\hat{n},\hat{s}\right\}$ on the grid, we then evaluate the test accuracy on held-out digits for the psMNIST task with RNN+*γ* networks with $\gamma (\cdot ;\hat{n},\hat{s})$ AF. Here we consider only the static homogeneous setting. We plot in Fig A-C and Fig A-D in S1 Appendix respectively the performance landscape for a fixed static activation, and the evolution of the parameters {*n*, *s*} as they are updated with gradient descent alongside training. The resulting performance landscapes are similar to what would be expected from the Jacobian-norm and Maximum Lyapunov Exponent λ_1_; unit-norm jacobian, and the edge of chaos, is favorable. More details in Section B.2 in S1 Appendix. Therefore, we set our initialization prior to be *n* ∼ *N*(5, 2^2^*I*) and *s* = 0 for all further analysis.

#### Pytorch autograd implementation of gamma

We implement *γ*(*x*; *n*, *s*) as a Pytorch autograd Function with corresponding Pytorch Module.

To allow for activation function adaptation, we further include the activation parameters in the backpropagation algorithm. We do so by defining the gradient of *γ* with respect to the input and parameters. We can rewrite *γ*(*x*; *n*, *s*) as:

$$\begin{array}{c} \gamma \left(x;n,s\right)=\frac{\left(1-s\right)}{n}\gamma_{1}\left(nx\right)+s\sigma \left(nx\right) \end{array}$$ (8)

where *σ*(*x*) is the sigmoid activation function. With this notation, the partial derivatives of *γ* with respect to *x* (or total derivative), *n* and *s* are

$$\begin{array}{c} \gamma^{\prime }\left(x;n,s\right)=\frac{\partial }{\partial x}\gamma \left(x;n,s\right)=\left(1-s\right)\sigma \left(nx\right)+ns\sigma \left(nx\right)\left(1-\sigma \left(nx\right)\right) \end{array}$$ (9)

$$\begin{array}{c} \frac{\partial }{\partial n}\gamma \left(x;n,s\right)=\frac{1-s}{n}\left(x\sigma \left(nx\right)-\frac{\gamma_{1}\left(nx\right)}{n}\right)+sx\sigma \left(nx\right)\left(1-\sigma \left(nx\right)\right) \end{array}$$ (10)

$$\begin{array}{c} \frac{\partial }{\partial s}\gamma \left(x;n,s\right)=\sigma \left(nx\right)-\frac{\gamma_{1}\left(nx\right)}{n} \end{array}$$ (11)

### 4.4 Evaluation methods and metrics

To assess how the activation gain and saturation influence on signal propagation, we use three quantities:

#### Jacobian norm

The main mechanism leading to the well studied problem of exploding & vanishing gradients in backpropagation and BPTT happens when products of Jacobian matrices explode or vanish [41, 48]. We average the *L*^2^-norm of the derivative of Eq (1) with respect to $h_{t-1}∼U\left(-5,5\right)$. A mean **Jacobian norm (JN)** that is greater/less than one leads to exploding/vanishing gradients, respectively. An issue with this approximation is that the true mean depends on ***h***_***t***_’s invariant distribution, which changes with (*n*, *s*).

#### Lyapunov exponents

Developed in Dynamical Systems theory, Lyapunov exponents measure the exponential rate of expansion/contraction of state space along iterates. Let us define *F*: *X* → *X* to be a continuously differentiable function, and consider the discrete dynamical system (*F*, *X*, *T*) defined by

$$\begin{array}{c} x_{t+1}=F\left(x_{t}\right) \end{array}$$ (12)

for all *t* ∈ *T*, where *X* is the phase space, and *T* the time range. Let *x*_0_, *w* ∈ *X*, define

$$\begin{array}{c} \lambda \left(x_{0},w\right)≝\underset{m\to \infty }{\text{lim}}\;\frac{1}{m}\;\text{ln}\;\prod_{t=1}^{m}\frac{\|DF^{t}\left(x_{0}\right)\cdot w\|}{\left\|w\right\|} \end{array}$$ (13)

$$\begin{array}{c} =\underset{m\to \infty }{\text{lim}}\;\frac{1}{m}\sum_{t=1}^{m}\;\text{ln}\;\frac{\|DF^{t}\left(x_{0}\right)\cdot w\|}{\left\|w\right\|} \end{array}$$ (14)

Note that once *x*_0_ and *w* have been fixed, the quantity λ(*x*_0_, *w*) is intrinsic to the discrete dynamical system defined by *x*_*t*+1_ = *F*(*x*_*t*_). We call λ(*x*_0_, *w*) a **Lyapunov exponent** of the mentioned dynamical system. As outlined in Section D.2 in S1 Appendix, it can be shown that under regularity conditions on the map *DF*^*t*^(⋅), the Lyapunov exponents effectively do not depend in the perturbation vector *w*, hence we consider λ(*x*_0_) only. Intuitively, Lyapunov exponents are topological quantities intrinsic to the dynamical system that describe the average amount of instability along infinite time horizons. Now, a randomly chosen vector, has a non-zero projection in the direction of the **Maximal Lyapunov exponent (MLE)** with probability 1, and thus over time the effect of the other Lyapunov exponents will become negligible. This motivates taking the MLE as a way of measuring the overall amount of stability or instability of a dynamical system. (see Section D in S1 Appendix for a LE primer). As an asymptotic quantity, the MLE has been used to quantify ANN stability and expressivity [40, 54]. The MLE gives a better measurement of stability than the Jacobian norm above, although it requires more effort to approximate. A positive MLE indicates chaotic dynamics and can lead to exploding gradients, while a negative MLE leads to vanishing ones.

We numerically compute the Maximal Lyapunov Exponent λ_1_(***h***_0_) for our RNN systems in Eq (1) using a QR algorithm (as motivated in Section D.3 in S1 Appendix). The computation relies on the recurrent matrix *W*_*hh*_, the activation function *γ*(⋅; ***n***, ***s***), and is a function of the initial condition ***h***_0_. We average the value of λ_1_ across i.i.d. ***h***_0_ draws from the stationary distribution *π*(*ξ*) of the process under a step-drive perturbation of amplitude *ξ* ≥ 0. For *ξ* = 0, this is the perturbation-less setting. If one consider $\left\{n,s\right\}\equiv \{n_{\hat{t}}\left(\xi \right),s_{\hat{t}}\left(\xi \right)\}$ as the activation parameters setting by the ARUN at time $\hat{t}$ after onset under a drive *ξ* (as in Fig 4C and 4D) this effectively makes λ_1_ a function of only $\hat{t}$ and *ξ* for fixed *W*_*hh*_.

#### Order of fractional order filtering

We investigate ARU activity under the lens of fractional differentiation [2, 10, 34]. The mechanism is defined implicitly by the product in Fourier domain *f* of the transformed signal with the operator *H*_*α*_(*f*) = (2*iπf*)^*α*^

$$\begin{array}{c} r\left(t\right)=D^{\alpha }g\left(t\right)=(g*h^{\alpha })\left(t\right)\Leftrightarrow R\left(f\right)=H_{\alpha }\left(f\right)G\left(f\right) \end{array}$$ (15)

or can be thought of as a three step process,

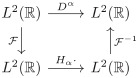

first transforming the signal *r* ∈ *L*^2^ in Fourier domain *R*(*f*), followed by a point-wise multiplication with *H*_*α*_(*f*), before finally performing an inverse Fourier transform back to the time domain. This procedure is how we perform fractional order differentiation for a signal, using the Discrete Fourier transform numpy.fft.fft (and associated fft.fftfreq and fft.ifft methods) from the Numpy [55] library. As noted in the main text, for a stimulus of the form *s*(*t*) = *ξ* ⋅ *H*(*t*) as studied in Section 2.3 where *ξ* ≥ 0 is the step size and *H*(*t*) is the Heaviside step function, a fractional differentiation (resp. integration) response *r*(*t*) = [*D*^*α*^*s*](*t*) would be proportional to *ξ* ⋅ *t*^−*α*^ for *α* > 0 (resp. *α* < 0).

To first determine whether an ARU signal *r*(*t*) can be explained by fractional differentiation, we perform curve fitting to the signals. Specifically in response to a step perturbation, we consider fitting exponential (*a* exp(−*t*/*b*) + *c*) and power law (*at*^−*b*^ + *c*) functions to the signal during the step perturbation. We fit curves over 100 time steps after onset of a step perturbation with *ξ* = 20.0. We evaluate goodness of fit by the mean squared error (MSE) between the curve fit and the signal. We standardize all signals by subtracting off their mean and dividing by the standard deviation—thus as a baseline, the MSE for a fit by a constant function (i.e. the mean) would be 1.0. For plotting the signals *r*(*t*) or reporting fit parameters, however, we report them on non-standardized signals.

Once we have established to consider fractional order differentiation, we leverage the procedure above to simultaneously estimate orders of fractional differentiation and obtain the fractionally differentiated signal. We determine the order *α* of fractional filtering by minimizing, over *α*, the mean square error (MSE) between the fractionally de-filtered *r*(*t*) activity and the original step perturbation. Specifically for *r*(*t*) the signal in time *t* in response to a step drive *s*(*t*), we determine $\hat{\alpha }$ as

$$\begin{array}{c} \hat{\alpha }=\underset{\alpha \in \left[-1,1\right]}{\text{arg}\;\text{min}}\;\|D^{-\alpha }r-s{\|}_{2}^{2} \end{array}$$ (16)

### 4.5 Network perturbations and task variations

To test the adaptive capabilities of our model and to compare it with conventional RNNs, we consider two different ways in which these external inputs may be perturbed:

1. **Variable contrast**: transforming the inputs *x*_*t*_. A brightness factor from a randomly sampled sinusoidal curve may multiplies the *x*_*t*_ input at each time-step *t* (Fig 2). These transformed inputs are then encoded by the same linear module *W*_*xh*_.
2. **Perturbed**: applying an external drive directly to the neurons. Taking inspiration from optogenetic stimulation, we inject a scalar external drive ξ∈R directly to the neuron (e.g. for ARUN, we add +*ξ* in Eq (3)), before the activation function is applied. This perturbation may either be a non-random scalar (Fig 2) or noisy.

## Supporting information

S1 Appendix A. Experimental details. **Fig A. A-B** Task independent stability metrics in activation parameter space. **C-D** Test accuracy in activation parameter space for the psMNIST task under two different learning scenarios. B. Performance: supplemental B.1 Comparison between homogeneous and heterogeneous activation functions. **Fig B**. Comparison between homogeneous and heterogeneous activation functions. Labels and perturbation details follow Fig 2. B.2 Further details on learning differences and performance in the static setting. B.3 Learned adaptation offers transfer learning advantages. **Fig C**. Trajectories of the activation parameters during retraining on the modified MNIST images. C. Adaptation: supplemental. C.1 Fractional differentiation. Fig D. (**Top**) Graph of a step to linear-increase function (right), then fractional order (*α* = 0.15) differentiated (left). (**Bottom**) Saturation *s*_*t*_ as a function of the time (left), for varying external drives *ξ* ∈ [0, 30] with the usual range applied during a stimulation period framed by the two dashed green lines. See next Fig E for colorbar. (right) The saturation signals *s*_*t*_ fractionally integrated with *α* = 0.15 reveal step to linear increase signals during the stimulation period. **Fig E**. Task: psMNIST. Random seed #: 400. Colorbar applies to whole figure. (**top-right**) mean ARU hidden-states for non-interacting ARUs, just as main text’s setting. For other panels, see respective titles. **Fig F**. Task: psMNIST. Random seed #: 500. Fig G. Task: sCIFAR10. Random seed #: 403. **Fig H**.**(a, c)** Distribution, over neurons, of fractional order *α* for interacting ARUs (**a**) and non-interacting, “Isolated”, ARUs (**c**). The order is established by minimizing the MSE between the fractional order differentiated signal of ARU activity and the step drive applied (*ξ* = 20 during *t* ∈ [100, 200)). If we apply the same procedure to the mean network activity, after averaging over neurons, we obtain the single estimate “*α* of mean resp” indicated by the black line. We report a Gaussian Kernel Density Estimate (KDE) of the distribution, with “scott” bandwidth estimation procedure. **(b, d)** Average activity for ARUs with the indicated fractional order *α*, for interacting ARUs (**b**) and non-interacting, “Isolated”, ARUs (**d**). C.2. Dynamic regularization. C.3. Sensitivity analysis for the perturbations experiments. **Fig I**. Sensitivity analysis for the step drive experiment. Lower is better. ARUN performs the best. **Fig J**. Sensitivity analysis for the Sinusoidal transformation on inputs, varying phase and amplitude alternatively. Lower is better. ARUN performs the best on average. **Fig K**. Sensitivity analysis for the noisy step drive experiment for the sCIFAR10 task. Higher is better. **Fig L**. Sensitivity analysis for the sinusoidal transformation on inputs, varying the frequency, for the sCIFAR10 task. Higher is better. C.4. Testing the evolutionary plausibility of our adaptive units. **Fig M**. Performance on the psMNIST classification task and robustness to the noise perturbed, step drive transformed and sine transformed inputs. The mean and standard deviation across three different initializations are shown. C.5. Gradient contribution according to position in input sequence. **Fig N**. Frobenius norm of the hidden-to-hidden weight matrix *W*_*hh*_ gradient contribution of a given input element, or pixel, as a function of that element’s position in the input sequence. The sequences are of length 784 and elements closer to position 0 are closer to the beginning of the input sequences. The gradients are computed in trained RNN+ReLU, RNN+*γ* heterogeneous and ARU networks on the psMNIST training set. Mean and standard deviation across three random initialization are shown. D. A primer on Lyapunov exponents. D.1. Definition of Lyapunov exponents. D.2. Oseledets theorem. D.3. The QR algorithm. D.4. Link to RNNs.
(PDF)
