# Supplementary material for: Neural networks with optimized single-neuron adaptation uncover biologically plausible regularization
Source: PLoS Comput Biol. 2024 Dec 13;20(12):e1012567. doi: 10.1371/journal.pcbi.1012567 (PMC11676530; doi:10.1371/journal.pcbi.1012567)
Supplement: S1 Appendix — A. Experimental details. Fig A. A-B Task independent stability metrics in activation parameter space. C-D Test accuracy in activation parameter space for the psMNIST task under two different learning scenarios. B. Performance: supplemental B.1 Comparison between homogeneous and heterogeneous activation functions. Fig B. Comparison between homogeneous and heterogeneous activation functions. Labels and perturbation details follow Fig 2. B.2 Further details on learning differences and performance in the static setting. B.3 Learned adaptation offers transfer learning advantages. Fig C. Trajectories of the activation parameters during retraining on the modified MNIST images. C. Adaptation: supplemental. C.1 Fractional differentiation. Fig D. (Top) Graph of a step to linear-increase function (right), then fractional order (α = 0.15) differentiated (left). (Bottom) Saturation st as a function of the time (left), for varying external drives ξ ∈ [0, 30] with the usual range applied during a stimulation period framed by the two dashed green lines. See next Fig E for colorbar. (right) The saturation signals st fractionally integrated with α = 0.15 reveal step to linear increase signals during the stimulation period. Fig E. Task: psMNIST. Random seed #: 400. Colorbar applies to whole figure. (top-right) mean ARU hidden-states for non-interacting ARUs, just as main text’s setting. For other panels, see respective titles. Fig F. Task: psMNIST. Random seed #: 500. Fig G. Task: sCIFAR10. Random seed #: 403. Fig H.(a, c) Distribution, over neurons, of fractional order α for interacting ARUs (a) and non-interacting, “Isolated”, ARUs (c). The order is established by minimizing the MSE between the fractional order differentiated signal of ARU activity and the step drive applied (ξ = 20 during t ∈ [100, 200)). If we apply the same procedure to the mean network activity, after averaging over neurons, we obtain the single estimate “α of mean resp” indicated by the black line. We report a [file pcbi.1012567.s001.pdf]

Supplementary Material for:
Neural networks with optimized single-neuron adaptation
uncover biologically plausible regularization

A Experimental details

Task independent stability metrics Fig. A shows the task-independent stability metrics of JN and MLE for a range of (n,s) values (fixed across neurons). We see that the activation shape influences Jacobian norms, thus confirming that it will play an important role during training. Consistent with the average gradient norm, the MLE reports distinct (n, s)-regions of stability for random networks. In some cases, expansion and contractions can be useful for computations, and we further use these measurements to study training dynamics.

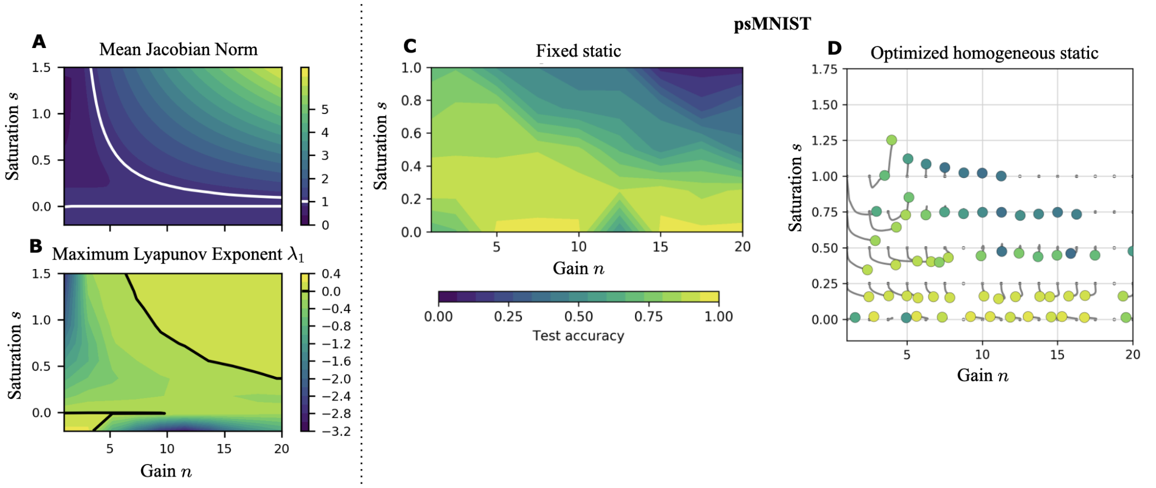

Figure A: **A-B** Task independent stability metrics in activation parameter space. **C-D** Test accuracy in activation parameter space for the psMNIST task under two different learning scenarios.

B Performance: supplemental

B.1 Comparison between homogeneous and heterogeneous activation functions

Within the optimized learning framework, where the activation parameters $\{n, s\}$ are learned by gradient descent of the loss, one could decide to enforce the activation function to be shared by all neurons (*homogeneous*) or vary neuron-to-neuron (*heterogeneous*). We incorporate this diversity by setting scalar $\{n, s\}$ parameters in the homogeneous case, and by setting vectors $\boldsymbol{n}, \boldsymbol{s} \in \mathbb{R}^N$ in the heterogeneous case for N neurons, such that the activation function of neuron i is set by $\boldsymbol{n}^i$, $\boldsymbol{s}^i$.

In terms of performance for the psMNIST task, we found that learning heterogeneous activation provided a slight increase but no significant advantages over the already well performing optimized homogeneous setting. On the gsCIFAR10 task the same conclusions hold, the heterogeneous RNN+γ performs as well if not slightly better than the homogeneous RNN+γ (see Fig. 2), however the increase in performance is again not statistically significant.

B.2 Further details on learning differences and performance in the static setting

As expected, we find a strong correlation between the norm of the Jacobian in parameter space which is task-independent (Fig. A**A**) and the performance landscapes for each task (see Fig. A**C**). Interestingly, regions in space (n, s) with poor performance are all associated with an exploding gradient, not a vanishing gradient. Networks whose activation functions have activation parameters in a neighborhood of $\{(n, s) : \|\gamma'(x; n, s)\| = 1\}$ present optimal performance, on all the tasks. On the one hand, this further emphasizes the performance of ReLU (see Ref [1]) as part of this (n, s)-neighborhood. However, as we show in Fig. A**C**-**D**, traditional

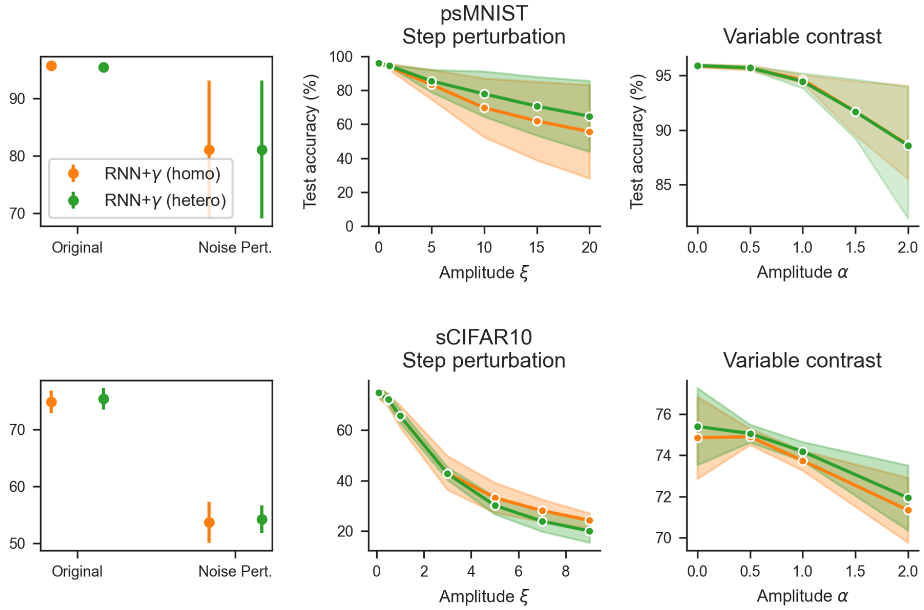

Figure B: Comparison between homogeneous and heterogeneous activation functions. Labels and perturbation details follow Fig. 2.

nonlinearities (including ReLU) are outperformed by the considerably different activation functions arising in the different scenarios of end-to-end learning. This result highlights that non trivial combinations of parameters may also achieve optimal performance while allowing for much more complex nonlinear transformations than ReLU.

B.3 Learned adaptation offers transfer learning advantages

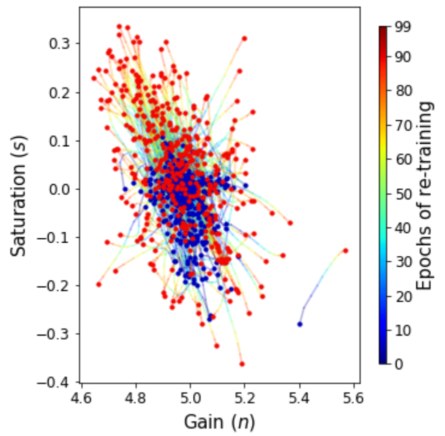

Figure C: Trajectories of the activation parameters during retraining on the modified MNIST images.

In neuroscience, the term adaptation is mostly used to describe processes that occur on short timescales and at a neuron level which have been shown to account for changes in stimulus statistics [2]. This mechanism is naturally linked to the concept of transfer learning in AI where one seeks systems where minimal changes in parameters allow adaptation from learned tasks to novel ones. To see if changes in single neurons activation could offer transfer advantages in ANNs, we design a novel task using the psMNIST test data set where the images are rotated by 45°. The goal is for a trained network to adapt to this change in input structure by only changing its activation function parameters. To evaluate this, we split rotated images into training and test sets, each containing approximately 5k images and the same number of images per digit. We then briefly retrain heterogeneous activation parameters (n_i, s_i) on this rotated data set using the heterogeneous adaptation scenario. For initialization, we take the parameters (including the (n_i, s_i)'s) that resulted from training with normal images, also under the heterogeneous adaptation scenario. Before retraining, the networks achieved an accuracy of 94% on the original data set, this fell to 42% after rotation. Retraining (n, s) allowed the networks to recover classification accuracy up to 56%. This shows that simply allowing the activation functions to adapt can recover over a quarter of lost performance (over 25%). An example of the variation of (n, s) trajectories after retraining is showed in Fig. 3**a** (bottom).

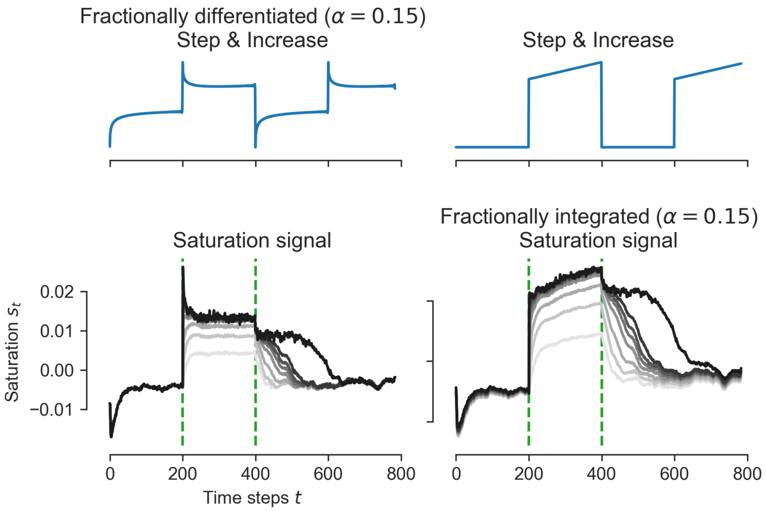

Figure D: (**Top**) Graph of a step to linear-increase function (right), then fractional order ($\alpha = 0.15$) differentiated (left). (**Bottom**) Saturation s_t as a function of the time (left), for varying external drives $\xi \in [0, 30]$ with the usual range applied during a stimulation period framed by the two dashed green lines. See next Fig. E for colorbar. (right) The saturation signals s_t fractionally integrated with $\alpha = 0.15$ reveal step to linear increase signals during the stimulation period.

Like in Fig. 3**a** (top), the cloud of (n, s) parameters expands with respect to its initialization, suggesting that a diversification in activation function shapes is needed to adapt to the change in task.

Allowing for small changes in the activation functions of individual neurons helps to mitigate the loss in performance caused by drastic changes in network inputs. The following question naturally arises from these results: is it possible to leverage the advantages brought by adaptation in an online manner instead of relying on retraining a part of the network? Such a "dynamic" adaptation process, which allows the network's activation functions to instantly change when presented with inputs of different statistics, would not only be less computationally expensive and faster but would also be more alike its natural counterpart. We further explore the idea of implementing rapid adaptation protocols for ANNs in the next section.

C Adaptation: supplemental

C.1 Fractional differentiation

Activation function parameters Further details on fractional order differentiation of the activation parameter signals, as opposed to the resulting hidden-states h_t, is included in Fig. D.

Determination of fractional order The order α of fractional order differentiation was determined as the arg-min (over α) of the mean square error between the fractional α-order integrated signal and the precise step inputs that drove the network. See Fig. E, and more details on the methodology can be found in Methods §5.4. We observe that this minimum is sharp, and observe close correspondence between the fractional order integrated signal and the original step-drive. This analysis was consistent across tasks and random seeds (see examples in Figs. E, F, G).

C.2 Dynamic regularization

Proposition 1. *For unitary W_{hh} weight initialization, the variance explained along a vector $u \in \mathbb{R}^{N_h}$ as a response to a perturbation $\boldsymbol{\eta} \sim \mathcal{N}(\boldsymbol{\mu}, \sigma^2 I)$ decays if and only if the parameters $\{\boldsymbol{n}_t, \boldsymbol{s}_t\}$ satisfy*

$$\sigma^2 \left[\frac{d}{dx} \gamma \left(x \, ; n_t^i, s_t^i \right) \Big|_{x=\mu^i} \right]^2 < 1 + \mathcal{O}\left(\sigma^3\right) \tag{1}$$

for $i \in \{1, \ldots, N_h\}$.

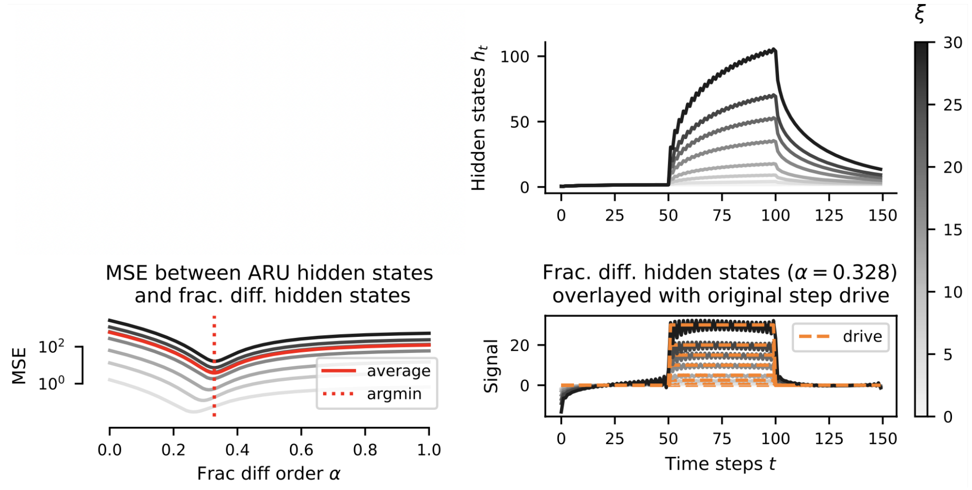

Figure E: Task: psMNIST. Random seed #: 400. Colorbar applies to whole figure. (**top-right**) mean ARU hidden-states for non-interacting ARUs, just as main text's setting. For other panels, see respective titles.

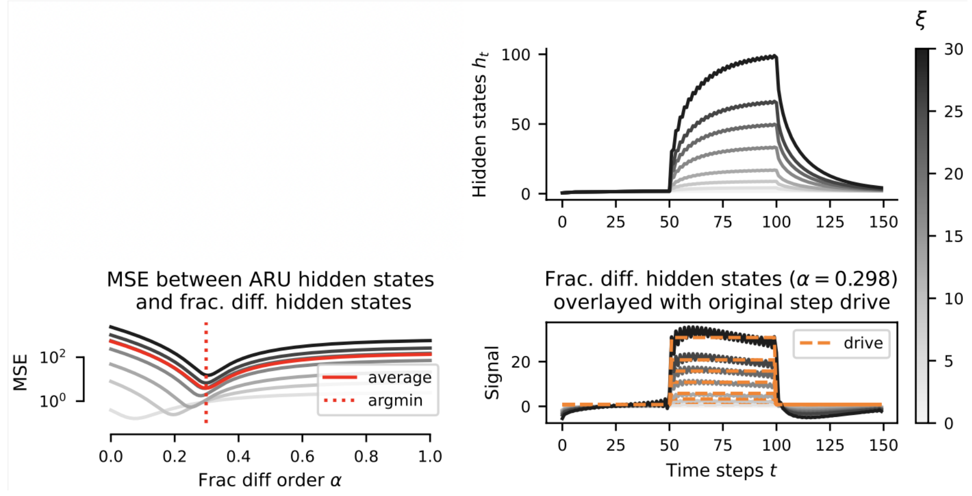

Figure F: Task: psMNIST. Random seed #: 500.

Proof. Consider some multivariate Gaussian noise $\eta \sim \mathcal{N}(\mu, \sigma^2 I)$, injected in the dynamics

$$h_t = \gamma\left(W_{hh} h_{t-1} + \eta\, ; n_t, s_t\right)$$

Now, the variance of this noise along a given vector $u \in \mathbb{R}^{N_h}$ as it propagates through the dynamics is given by:

$$\text{Var}\left[u^\top W_{hh} \gamma(\eta\, ; n_t, s_t)\right] = u^\top W_{hh} \text{Var}\left[\gamma(\eta\, ; n_t, s_t)\right] W_{hh}^\top u$$

after one iteration. Since η is chosen such that η_i is independent of η_j for $i \neq j$, i.e. $\text{Cov}[\eta_i, \eta_j] = \sigma^2 \delta_{ij}$, and $\gamma(\cdot)$ acts element-wise, we have that $\text{Cov}\left[\gamma(\eta_i), \gamma(\eta_j)\right] = 0$. As such,

$$\text{Var}\left[\gamma(\eta\, ; n_t, s_t)\right] = \text{diag}\left\{\text{Var}\left[\gamma(\eta_i; n_t^i, s_t^i)\right]\right\}_{1 \leq i \leq n} =: D_{n,s}$$

and

$$\text{Var}\left[u^\top W_{hh} \gamma(\eta\, ; n, s)\right] = u^\top W_{hh} D_{n,s} W_{hh}^\top u \tag{2}$$

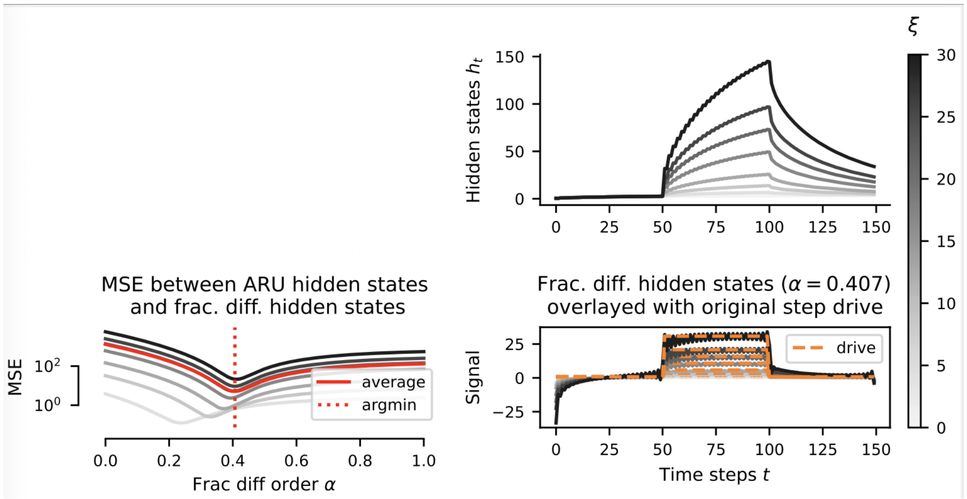

Figure G: Task: sCIFAR10. Random seed #: 403.

Using a first order Taylor expansion of $\gamma(x\,; n_t, s_t)$ about the mean of η, we obtain an approximation of the variance

$$[D_{n,s}]_{i,i} = \mathrm{Var}\left[\gamma(\eta_i; n_t^i, s_t^i)\right] = \sigma^2 \left[\frac{d}{dx}\gamma\left(\mathbb{E}[\eta_i]; n_t^i, s_t^i\right)\right]^2 + O\left(\mathbb{E}\left[(\eta_i - \mathbb{E}[\mu_i])^3\right]\right) \tag{3}$$

$$= \sigma^2 \left[\frac{d}{dx}\gamma\left(\mathbb{E}[\eta_i]; n_t^i, s_t^i\right)\right]^2 + O\left(\sigma^3\right) \tag{4}$$

where the first term of the RHS can easily be evaluated directly (see equation (9) for a closed form expression of $\frac{d}{dx}\gamma$). Also, under the initialization schemes considered in our experiments, W_{hh} is unitary and as such $W_{hh}D_{n,s}W_{hh}^\top$ defines a normal matrix with eigenvalues exactly given by the entries of diagonal matrix $D_{n,s}$. This gives the result. □

This result reformulates known conditions on an RNN's Jacobian and indicates that under mild connectivity assumptions, the left hand side of must remain smaller than one to avoid noise amplification. For example, for a linear AF with slope a, we would require $\sigma \leq 1/a$.

Parameter evolution for noise integration Let us for a moment restrict our attention to a single neuron, thus removing subscripts i and assuming scalar quantities. We note in passing that σ is non-zero even for scalar ξ as our formalization accounts for the linearly scaled inputs x_t, which are distributed under the task input statistics. Now, consider the level set

$$\Lambda(\xi) := \left\{(n, s) : \frac{\partial}{\partial x}\gamma\left(\mu + \xi\,; n, s\right) = \frac{1}{|\sigma|}\right\} \tag{5}$$

consisting of (n, s) values at the boundary of the region derived from Proposition 1 for a noise shifted by an external drive $\xi \geq 0$ (un-perturbed if $\xi = 0$). As mentioned earlier this set corresponds to a manifold in (n, s) space, one that shifts as a function of ξ (see Fig. 5**b** for a visualization of these curves). Take $\{\hat{n}, \hat{s}\}$ satisfying Prop. 1, and assume that there exists $\epsilon > 0$ for which $d(\{\hat{n}, \hat{s}\}, \Lambda(0)) = \epsilon$. For noise robustness to be maintained in stimulated regimes, we have that the activation parameters $\{n(\xi), s(\xi)\}$ should shift to stay within the region highlighted by Prop 1, i.e. $d(\{\hat{n}, \hat{s}\}, \Lambda(\xi)) > 0$ for all $\xi \geq 0$. This is what we observe, see Fig. 5**b**. By fixing this distance and given an initial condition $\{n_0, s_0\}$, one can solve the above system to obtain a path $\{n(\xi), s(\xi)\}$ in parameter space as a function of ξ (assuming continuous dependence on ξ). This path corresponds to the expected variation in activation parameters $\{n, s\}$ as a function of ξ for the system to absorb, through the hidden-state dynamics, the injected noise by a margin ϵ.

Still, this does not account for particular behavior observed of an onset value $\{n_0, s_0\}$ decreasing or increasing with an exponential time-constant to a steady-state value $\{n_\infty, s_\infty\}$, in a matter akin to spike frequency adaptation. Both onsets and steady-states satisfy the observations previously highlighted, but their–distinct–existence is unaccounted for. This sets a rich ground for future work.

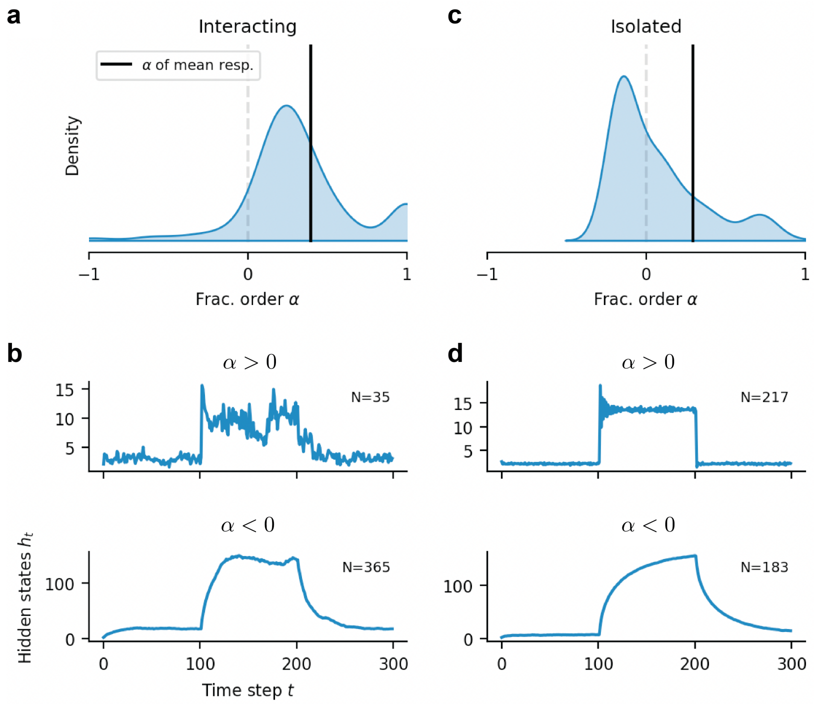

Figure H: **(a, c)** Distribution, over neurons, of fractional order α for interacting ARUs (**a**) and non-interacting, "Isolated", ARUs (**c**). The order is established by minimizing the MSE between the fractional order differentiated signal of ARU activity and the step drive applied ($\xi = 20$ during $t \in [100, 200)$). If we apply the same procedure to the mean network activity, after averaging over neurons, we obtain the single estimate "α of mean resp" indicated by the black line. We report a Gaussian Kernel Density Estimate (KDE) of the distribution, with "scott" bandwidth estimation procedure. **(b, d)** Average activity for ARUs with the indicated fractional order α, for interacting ARUs (**b**) and non-interacting, "Isolated", ARUs (**d**).

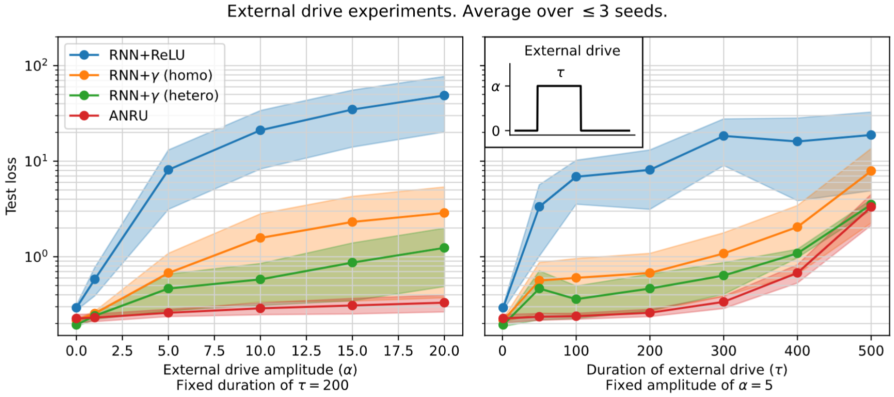

Figure I: Sensitivity analysis for the step drive experiment. Lower is better. ARUN performs the best.

C.3 Sensitivity analysis for the perturbations experiments

We refer to Figs. I-L.

C.4 Testing the evolutionary plausibility of our adaptive units

The performance and robustness results presented in the main paper were obtained by randomly initializing and then simultaneously training both the main RNN networks as well as the adaptive sub-networks. For our

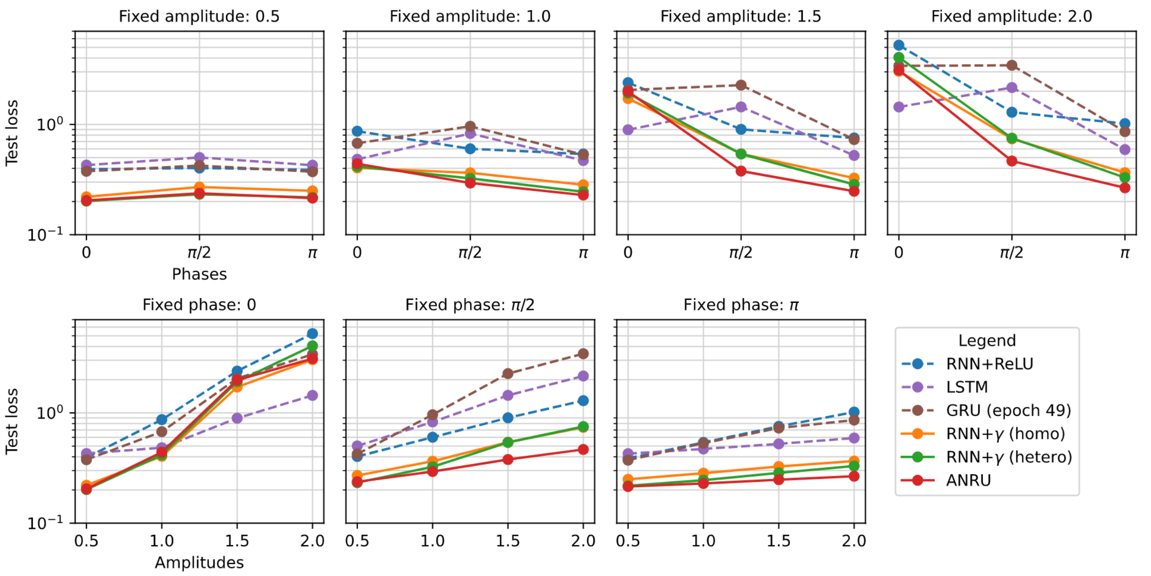

Figure J: Sensitivity analysis for the Sinusoidal transformation on inputs, varying phase and amplitude alternatively. Lower is better. ARUN performs the best on average.

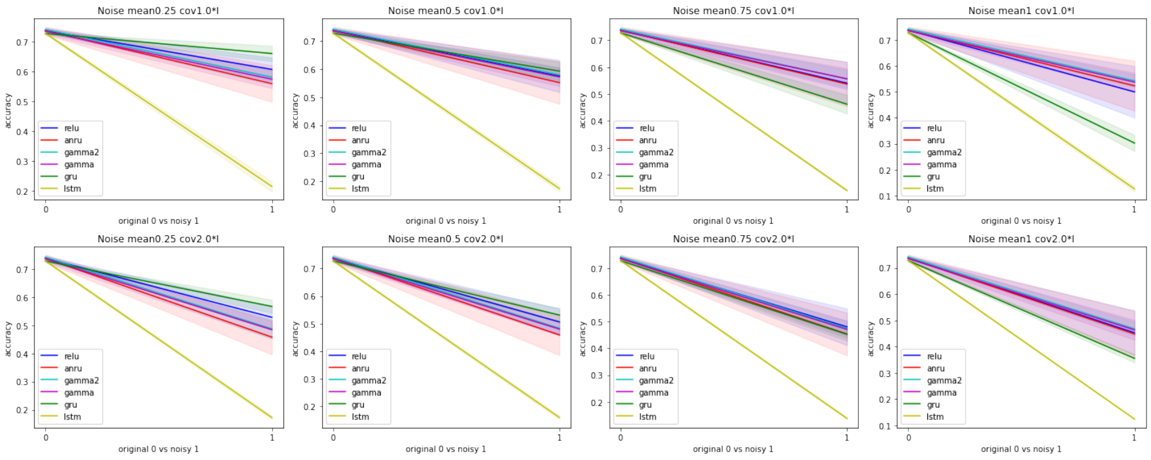

Figure K: Sensitivity analysis for the noisy step drive experiment for the sCIFAR10 task. Higher is better.

adaptive units to adequately model adaptation in biological neurons, the adaptive sub-units of each network should in principle be fixed when training the main RNN network. Indeed, in the brain, single neuron adaptation mechanisms have been developed over evolutionary timescales and are passed down through genetic information.

In this section we test our AURNs in a more biologically plausible setting, and see if the structure of the adaptive sub-network can be efficiently passed down from a network to another without affecting the network's performance or robustness. To verify this we have tested the performance and robustness to the noise, step and sine data transformations of AURNs generated using two distinct initializing and training scenarios:

- **Scenario 1:** Both the main and the adaptive RNNs are randomly initialized, using a specified random seed (here denoted $seed_1$), and trained from scratch as previously described. All results from the paper are obtained with AURNs generated using this scenario.
- **Scenario 2:** The main RNN is randomly initialized using a specified random seed ($seed_2$) while the adaptive sub-network is taken from a scenario 1 trained AURN with $seed_1 \neq seed_2$. The main RNN is then trained using the same training procedure as in scenario 1 but the adaptive sub-network's parameters are kept constant.

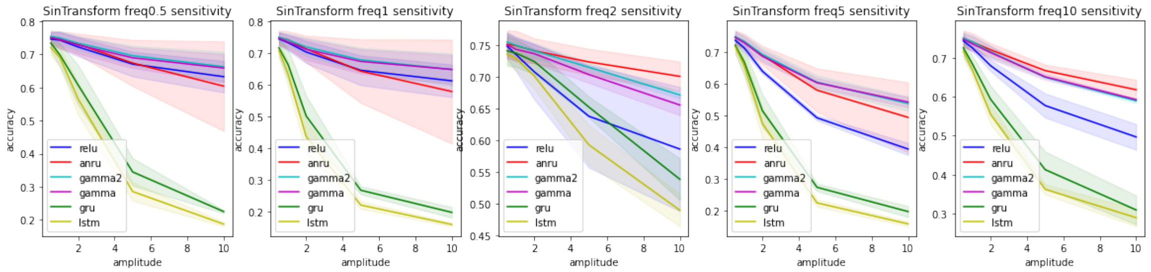

Figure L: Sensitivity analysis for the sinusoidal transformation on inputs, varying the frequency, for the sCIFAR10 task. Higher is better.

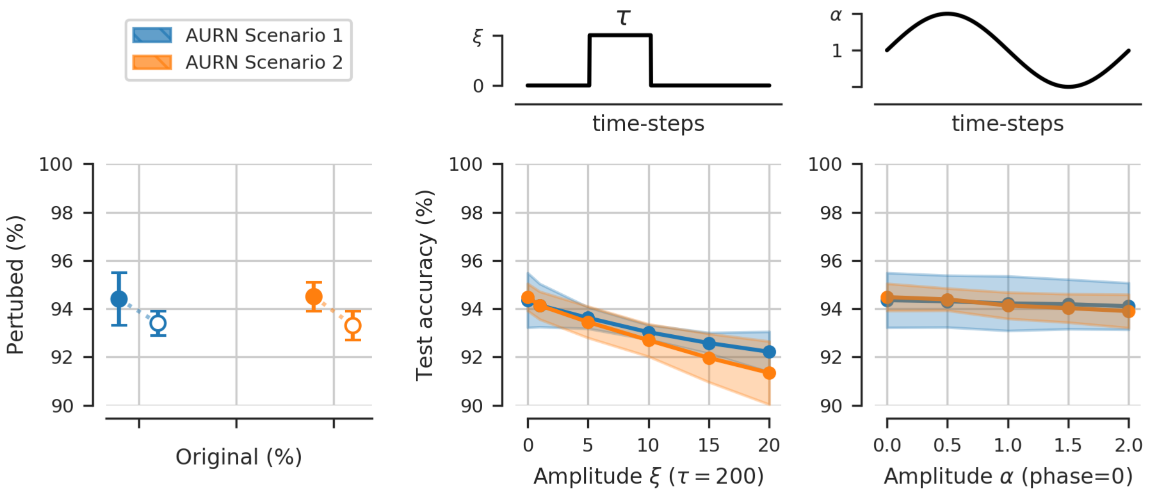

Figure M: Performance on the psMNIST classification task and robustness to the noise perturbed, step drive transformed and sine transformed inputs. The mean and standard deviation across three different initializations are shown.

This was done for multiple random seeds of both the main RNN and the trained adaptive RNN, the results are shown in Fig. M. We can see that the adaptation mechanisms previously learned with a specific main network can be used, as efficiently, by another main network without needing any re-training of the sub-network. The performance and robustness to different perturbations are, for all practical purposes, the same in both the setting where the main and the adaptive networks were trained simultaneously (scenario 1) and the setting where the adaptive sub-network was imported from a previously trained network and only the main network was trained (scenario 2).

C.5 Gradient contribution according to position in input sequence

The well documented vanishing and exploding gradients problems of RNNs prohibit effective training over long timescales. In particular, the gradient of the loss computed with the output of the network at time-step $t + \delta$ with respect to the hidden states of the network at time-step t either decays or increases exponentially with δ. In the vanishing case this makes the learning of long term dependencies impossible, while in the exploding case the entire training procedure is compromised. This phenomenon is linked to dynamic regimes in which an RNN operates, and is thus related to the leading Lyapunov exponent measurement described above (see Refs [3, 4] for more details).

We quantify the effects of learned neural adaptation on gradient propagation in RNNs by computing the gradient norms of the hidden-to-hidden weight matrix (W_{hh} in equations 1 and 3) on the psMNIST training set starting the gradient accumulation at different points in the input sequence. This was done for trained networks to take into consideration the learned adaptive behavior when considering the gradient propagation. In RNN+ReLU networks and to some lesser extend in RNN+γ heterogeneous networks the gradient norm

increases monotonously with sequence length, as shown in logarithmic scale in Fig. N. The earlier the accumulation of the gradients is started for these two network types the larger their norm is at the end of the input sequence when the loss is computed. In ARU networks however, after an initial increase the norms of the gradients actually decrease with sequence length. When the gradients are computed using the entire input sequences, the norm of the W_{hh} gradients in ARU networks is an order of magnitude smaller than in RNN+ReLU or in RNN+γ heterogeneous networks which promotes trainability and the stability of the gradient propagation during the training procedure. We also note that in ARU networks, elements (pixels) which are at the beginning of the input sequence and further away from the moment the loss is computed actually contribute more to the gradient of the weights when compared to later inputs. This is in stark contrast with gradient contribution in RNN+ReLU networks where the gradient contribution is monotonously increasing with the element's position in the input sequence, early inputs contributing much less than later inputs to the gradient.

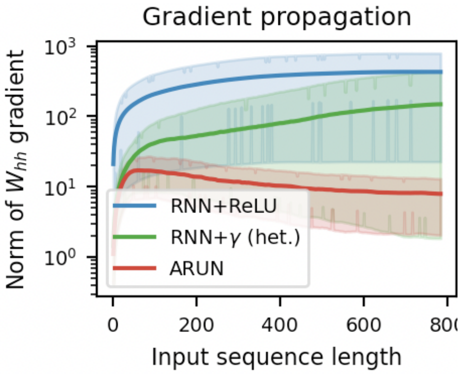

Figure N: Frobenius norm of the hidden-to-hidden weight matrix W_{hh} gradient contribution of a given input element, or pixel, as a function of that element's position in the input sequence. The sequences are of length 784 and elements closer to position 0 are closer to the beginning of the input sequences. The gradients are computed in trained RNN+ReLU, RNN+γ heterogeneous and ARU networks on the psMNIST training set. Mean and standard deviation across three random initialization are shown.

D A primer on Lyapunov exponents

In this section we are first going to give a bit of theoretical background on Lyapunov exponents. Exponential explosion and vanishing of long products of Jacobian matrices is a long studied topic in dynamical systems theory, where an extensive amount of tools have been developed in order to understand these products. Thus one can hope to leverage these tools in order to better understand the exploding and vanishing gradient problem in the context of RNNs.

D.1 Definition of Lyapunov exponents

Let $F : X \to X$ be a continuously differentiable function, and consider the discrete dynamical system (F, X, T) defined by

$$x_{t+1} = F(x_t) \tag{6}$$

for all $t \in T$, where X is the phase space, and T the time range. We would like to gain an intuition for how trajectories of the mentioned dynamical system behave under small perturbations.

Let x_t and x'_t be two trajectories with initial conditions x_0 and x'_0, such that $|x_0 - x'_0|$ is sufficiently small.

Defining $\epsilon_t = x'_t - x_t$, we get by the first order Taylor expansion

$$\begin{aligned} x'_{t+1} &= F(x'_t) && (7)\\ &= F(x_t + \epsilon_t) && (8)\\ &= F(x_t) + DF(x_t) \cdot \epsilon_t + O(|\epsilon_t|^2) && (9)\\ &= x_{t+1} + DF(x_t) \cdot \epsilon_t + O(|\epsilon_t|^2) && (10) \end{aligned}$$

Substracting x_{t+1} both sides we get the variational equation

$$\begin{aligned} \epsilon_{t+1} &= DF(x_t) \cdot \epsilon_t + O(|\epsilon_t|^2) && (11)\\ &\approx \prod_{k=0}^{t} DF(x_k) \cdot \epsilon_0 && (12)\\ &= DF^{t+1}(x_0) \cdot \epsilon_0 && (13) \end{aligned}$$

(Here $DF^{t+1}(x_0)$ is an abuse of notation for the Jacobian of the $(t+1)$-th iterate of F, evaluated at x_0). Intuitively the ratio $\frac{\|\epsilon_t\|}{\|\epsilon_0\|} = \frac{\|DF^t(x_0)\cdot\epsilon_0\|}{\|\epsilon_0\|}$ describes the expansion/contraction rate after t time steps if our initial perturbation was ϵ_0, which motivates the following definition:

Let $x_0, w \in X$, define

$$\lambda(x_0, w) \stackrel{\text{def}}{=} \lim_{m\to\infty} \frac{1}{m} \ln \prod_{t=1}^{m} \frac{\|DF^t(x_0)\cdot w\|}{\|w\|} \tag{14}$$

$$= \lim_{m\to\infty} \frac{1}{m} \sum_{t=1}^{m} \ln \frac{\|DF^t(x_0)\cdot w\|}{\|w\|} \tag{15}$$

Thus $\lambda(x_0, w)$ measures the average rate of expansion/contraction over an infinite time horizon of the trajectory starting at x_0, if it has been given an initial perturbation w. Note that once x_0 and w have been fixed, the quantity $\lambda(x_0, w)$ is intrinsic to the discrete dynamical system defined by $x_{t+1} = F(x_t)$. We call $\lambda(x_0, w)$ a **Lyapunov exponent** of the mentioned dynamical system.

Since the Lyapunov exponents describe the the average rate of expansion/contraction for long products of Jacobian matrices, it doesn't sound too surprising that they might provide an interesting perspective to study the exploding and vanishing gradient problem in RNNs. To give a complete picture of the analogy to RNNs, one can think of x_t as the hidden state at time t, and F can be seen as the function defined in the RNN cell. The only difference is that in RNNs we have inputs at every time steps, and thus the function F changes at every time step. This is the distinction between autonomous and non-autonomous dynamical systems, which is explained in more detail in the upcoming subsection D.4.

Finally, let us remark that the expression in the above definition of Lyapunov exponents is not always well defined. This will be the topic of the next subsection D.2, where we are presenting Oseledets theorem which gives exact conditions for when the above expression in well-defined.

D.2 Oseledets theorem

As already stated, we bypassed the fact that the limit in the definition of $\lambda(x_0, w)$ might not actually exists. In fact this is the result of the well-known *Oseledets theorem*, but before stating the theorem let us point out a definition.

Definition. A *cocycle* of an autonomous dynamical system (F, X, T) is a map $C : X \times T \to \mathbb{R}^{n\times n}$ satisfying:

- $C(x_0, 0) = \text{Id}$
- $C(x_0, t+s) = C(x_t, s)C(x_0, t)$ for all $x_0 \in X$ and $s, t \in T$

Oseledets theorem. (sometimes referred to as Oseledets *multiplicative ergodic theorem*) Let μ be an ergodic invariant measure on X, and let C be a cocycle of a dynamical system (F, X, T) such that for each $t \in T$, the maps $x \mapsto \log\|C(x,t)\|$ and $x \mapsto \log\|C(x,t)^{-1}\|$ are L^1-integrable with respect to μ. Then for μ-almost all x and each non-zero vector $w \in \mathbb{R}^n$ the limit

$$\lambda(x, w) = \lim_{t\to\infty} \frac{1}{t} \ln \frac{\|C(x,t)w\|}{\|w\|} \tag{16}$$

exists and assumes, depending on w but not on x, up to n different values, called the Lyapunov exponents (giving rise to a more general definition)

One can prove that the following matrix limit

$$\Lambda = \lim_{t\to\infty} [C(x,t)^T C(x,t)]^{1/2t} \tag{17}$$

exists, is symmetric positive-definite and its log-eigenvalues are the Lyapunov exponents. We call Λ the *Oseledets matrix.*

In order to make this definition a little bit more intuitive, let us come back to our original situation, and note that the terms $\prod_{k=0}^{t} DF(x_k) = DF^{t+1}(x_0)$ define a cocycle verifying the conditions of the theorem. Thus,

in this case, the Lyapunov exponents are not only well defined, but there are up to n distinct ones of them, and they are the log-eigenvalues of the following Oseledets matrix:

$$\Lambda = \lim_{t\to\infty} [DF^t(x_0)^T \cdot DF^t(x_0)]^{1/2t} \tag{18}$$

Let us now consider the singular value decomposition of $DF^t(x_0)$,

$$DF^t(x_0)V(x_0,t) = U(x_0,t)\Sigma(x_0,t) \tag{19}$$

where $\Sigma(x_0,t)$ is a diagonal matrix composed of the singular values $\sigma_1(x_0,t) \geq \ldots \geq \sigma_n(x_0,t) \geq 0$, and $U(x_0,t)$ as well as $V(x_0,t)$ are orthogonal matrices, composed column-wise of the left and right singular vectors respectively. Then

$$\Lambda = \lim_{t\to\infty} V(x_0,t)^T \Sigma(x_0,t)^{1/t} V(x_0,t) \tag{20}$$

Thus, for large t, the log-eigenvalues of Λ can be approximated by $\frac{1}{t}\ln\sigma_i(x_o,t)$'s, which can be thought of as the average singular value along an infinite time horizon. It turns out that for ergodic systems, the Lyapunov exponents are independent of initial conditions x_0. Thus, intuitively, Lyapunov exponents are topological quantities intrinsic to the dynamical system that describe the average amount of instability along infinite time horizons.

In order to understand how this instability manifests along each direction, let us further look what we can say about the vectors associated with the individual Lyapunov exponents. If we denote $\lambda^{(1)} \geq \lambda^{(2)} \geq \ldots \geq \lambda^{(s)}$ the *distinct* Lyapunov exponents, and $v_i(x_0)$ the corresponding vector of the matrix $\lim_{t\to\infty} V(x_0,t)$, then let us define the nested subspaces

$$S_j(x_0) = \text{span}\{v_i(x_0) | i = j, j+1, \ldots, s\} \tag{21}$$

for all $j = 1, 2, \ldots, s$, and take a vector $w_j(x_0) \in S_j(x_0) \setminus S_{j+1}(x_0)$. Then $w_j(x_0)$ is orthogonal to all $v_i(x_0)$ with $i < j$, and has a non-zero projection onto $v_j(x_0)$ since $v_j(x_0) \notin S_{j+1}(x_0)$, and thus

$$\|DF^t(x_0) \cdot w_j(x_0)\| \sim e^{\lambda^{(j)}t} \tag{22}$$

In particular, since $S_1(x_0)$ is the whole phase space X, and $S_2(x_0)$ is only a hyperplane in X (a subset of Lebesgue measure zero), we have that for "almost all" $w \in X$:

$$\|DF^t(x_0) \cdot w\| \sim e^{\lambda^{(1)}t} \tag{23}$$

hence aligning with the direction of maximum Lyapunov exponent (MLE). In other words a randomly chosen vector, has a non-zero projection in the direction of the MLE with probability 1, and thus over time the effect of the other exponents will become negligible. This motivates taking the MLE as a way of measuring the overall amount of stability or instability of a dynamical system. One typically distinguishes the cases, where the MLE is negative, zero and positive.

Thus computing MLEs, LEs and their corresponding subspaces can be a useful tool to understand the average expansion/ contraction rate as well as the corresponding directions of gradients in recurrent neural networks.

D.3 The QR algorithm

It is generally not advised to calculate the Lyapunov exponents and the associated vectors using $DF^t(x_0)$ as this matrix becomes increasingly ill-conditioned. There is a known algorithm that in most cases allows to provide good estimates, called the *QR algorithm*.

As a preliminary remark, let us emphasize that the right singular vectors of $DF(x_{t+1})$ do not necessarily match the left singular vectors of $DF(x_t)$, thus simply applying the singular value decomposition in order to calculate the Lyapunov exponents does not work.

Let us denote $J_t = DF(x_t)$ for each time step $t = 0, 1, 2, \ldots$, then lets us pick an orthogonal matrix Q_0, and compute $Z_0 = J_0 Q_0$. Then let us perform the QR decomposition $Z_0 = Q_1 R_1$. Let us further assume that J_0 is invertible and we are imposing that the diagonal elements of R_1 are non-negative (which we can), thus making the QR decomposition unique.

In the next step, we compute $Z_1 = J_1 Q_1$ and perform the QR decomposition $Z_1 = Q_2 R_2$, where again we are imposing the diagonal elements of R_2 to be non-negative.

Continuing in this fashion at each time step k, we then have the identity $J_k = Q_{k+1} R_{k+1} Q_k^T$, and thus

$$DF^{t+1}(x_0) = \prod_{k=0}^{t} J_k \tag{24}$$

$$= Q_{t+1}(R_t \cdot \ldots \cdot R_1) Q_0^T \tag{25}$$

It turns out that, as long as the dynamical system is "regular", we can then compute the i-th Lyapunov exponent via

$$\lambda_i = \lim_{t \to \infty} \frac{1}{t} \sum_{k=1}^{t} \ln(R_k)_{ii} \tag{26}$$

where the Lyapunov exponents are ordered $\lambda_1 \geq \lambda_2 \geq \ldots \geq \lambda_n$ as explained in Ref [5] and Ref [6].

D.4 Link to RNNs

Recalling the update equation of an RNN:

$$h_{t+1} = \phi(V h_t + U x_{t+1} + b) \tag{27}$$

for $t = 0, 1, \ldots$, and by denoting $F(h, x) = \phi(Vh + Ux + b)$, we can see that

$$\tilde{h}_{t+1} = F(\tilde{h}_t, 0) \tag{28}$$

defines an autonomous discrete dynamical system (DS1), while

$$h_{t+1} = F(h_t, x_{t+1}) \tag{29}$$

defines a non-autonomous discrete dynamical system (DS2).

For (DS1), the machinery that we have developed over the last subsections is directly applicable, as we are in the autonomous case. For instance, we can compute the Lyapunov exponents of recurrent neural network over the course of training using the QR algorithm, and in particular observe the evolution of the maximum Lyapunov exponent (MLE), as a means to measure the amount of instability or chaos in the network. For example in the case of a linear RNN with a unitary or orthogonal connectivity matrix, all LEs are equal to zero, and thus no expansion nor contraction is happening. If all LEs are negative, we are in the contracting regime, where every point eventually will approach an attractor, thus producing a vanishing gradient. For instance, Ref [7] showed that storing information in a fixed-size state vector (as is the case in a vanilla RNN) over sufficiently long time horizon in a stable way necessarily leads to vanishing gradients when back-propagating through time (here stable means insensitive to small input perturbations).

The natural question arises whether and to what extent the machinery will stay valid for (DS2). It turns out that one can use the theory of Random Dynamical Systems Theory, where Oseledet's multiplicative ergodic theorem holds under some stationarity assumption of the underlying distribution generating the inputs x_t as stated in Ref [8]. However in this paper we are just making use of the machinery developed for (DS1), by computing Lyapunov exponents for trained RNNs but computed without inputs ($x_t = 0$ for all t).

Supplementary References

References

[1] Xavier Glorot, Antoine Bordes, and Yoshua Bengio. Deep sparse rectifier neural networks. In Geoffrey Gordon, David Dunson, and Miroslav Dudík, editors, *Proceedings of the Fourteenth International*

Conference on Artificial Intelligence and Statistics, volume 15 of *Proceedings of Machine Learning Research*, pages 315–323, Fort Lauderdale, FL, USA, 11–13 Apr 2011. PMLR.

[2] Alison I. Weber, Kamesh Krishnamurthy, and Adrienne L. Fairhall. Coding principles in adaptation. *Annual Review of Vision Science*, 5(1):427–449, 2019. PMID: 31283447.

[3] Ryan Vogt, Maximilian Puelma Touzel, Eli Shlizerman, and Guillaume Lajoie. On lyapunov exponents for rnns: Understanding information propagation using dynamical systems tools. *Frontiers in Applied Mathematics and Statistics*, 8, 2022.

[4] B. Poole, S. Lahiri, M. Raghu, J. Sohl-Dickstein, and S. Ganguli. Exponential expressivity in deep neural networks through transient chaos. *arXiv e-prints*, June 2016.

[5] Giancarlo Benettin, L Galgani, Antonio Giorgilli, and Marie Strelcyn. Lyapunov characteristic exponents for smooth dynamical systems and for hamiltonian systems; a method for computing all of them. part 1: theory. *Meccanica*, 15:9–20, 03 1980.

[6] Luca Dieci and Erik S. Van Vleck. Computation of a few lyapunov exponents for continuous and discrete dynamical systems. *Applied Numerical Mathematics*, 17(3):275 – 291, 1995. Special Issue on Numerical Methods for Ordinary Differential Equations.

[7] Y Bengio, P Simard, and P Frasconi. Learning long-term dependencies with gradient descent is difficult. *IEEE Transactions on Neural Networks*, 5(2):157–166, 1994.

[8] Ludwig Arnold. *Random Dynamical Systems*. Springer, 1998.
